# Ocular macrophage origin and heterogeneity during steady state and experimental choroidal neovascularization

**DOI:** 10.1186/s12974-020-02010-0

**Published:** 2020-11-13

**Authors:** Steven Droho, Benjamin R. Thomson, Hadijat M. Makinde, Carla M. Cuda, Harris Perlman, Jeremy A. Lavine

**Affiliations:** 1 Department of Ophthalmology, Feinberg School of Medicine, Northwestern University, 240 E Huron St, McGaw M343, Chicago, IL 60611 USA; 2 Department of Medicine, Division of Nephrology and Hypertension, Feinberg Cardiovascular and Renal Research Institute, Feinberg School of Medicine, Northwestern University, Chicago, IL USA; 3 Department of Medicine, Division of Rheumatology, Feinberg School of Medicine, Northwestern University, Chicago, IL USA

**Keywords:** Age-related macular degeneration (AMD), Choroidal neovascularization (CNV), Angiogenesis, Macrophage

## Abstract

**Background:**

Neovascular age-related macular degeneration (nAMD) commonly causes vision loss from aberrant angiogenesis, termed choroidal neovascularization (CNV). Macrophages are heterogeneous cells that are necessary for experimental CNV, present in human CNV samples, and can display diverse functions, which are dependent upon both their origin and tissue microenvironment. Despite these associations, choroidal macrophage heterogeneity remains unexplored.

**Methods:**

We performed multi-parameter flow cytometry on wildtype (WT) and *Ccr2*^−/−^ mice after laser injury to identify macrophage subtypes, and determine which subsets originate from classical monocytes. To fate map tissue resident macrophages at steady state and after laser injury, we used the *Cx3cr1*^*CreER/+*^ ; *Rosa26*^*zsGFP/+*^ mouse model. We reanalyzed previously published single-cell RNA-seq of human choroid samples from healthy and nAMD patients to investigate human macrophage heterogeneity, disease association, and function.

**Results:**

We identified 4 macrophage subsets in mice: microglia, MHCII^+^CD11c^−^, MHCII^+^CD11c^+^, and MHCII^−^. Microglia are tissue resident macrophages at steady state and unaffected by laser injury. At steady state, MHCII^−^ macrophages are long lived, tissue resident macrophages, while MHCII^+^CD11c^−^ and MHCII^+^CD11c^+^ macrophages are partially replenished from blood monocytes. After laser injury, MHCII^+^CD11c^−^ macrophages are entirely derived from classical monocytes, MHCII^−^ macrophages originate from classical monocytes (90%) and an expansion of tissue resident macrophages (10%), and MHCII^+^CD11c^+^ macrophages are derived from classical monocytes (70%), non-classical monocytes (10%), and an expansion of tissue resident macrophages (20%). Single-cell RNA-seq analysis of human choroid found 5 macrophage subsets: two MHCII^+^CD11C^−^ and three MHCII^+^CD11C^+^ populations. One MHCII^+^CD11C^+^ subset was 78% derived from a patient with nAMD. Differential expression analysis identified up-regulation of pro-angiogenic gene expression in one MHCII^+^CD11C^−^ and two MHCII^+^CD11C^+^ subsets, including the disease-associated cluster. The upregulated MHCII^+^CD11C^−^ pro-angiogenic genes were unique compared to the increased MHCII^+^CD11C^+^ angiogenesis genes.

**Conclusions:**

Macrophage origin impacts heterogeneity at steady state and after laser injury in mice. Both mice and human patients demonstrate similar macrophage subtypes. Two discrete pro-angiogenic macrophage populations exist in the human choroid. Targeting specific, pro-angiogenic macrophage subsets is a potential novel therapeutic for nAMD.

**Supplementary Information:**

The online version contains supplementary material available at 10.1186/s12974-020-02010-0.

## Background

Age-related macular degeneration (AMD) is the most common cause of vision loss in the developed world. AMD exists in two forms: non-neovascular/dry AMD and neovascular/wet AMD (nAMD). Non-neovascular AMD develops when inflammatory lipoprotein deposits called drusen accumulate under the retinal pigment epithelium (RPE). nAMD occurs when angiogenesis from the choroidal vasculature, theoretically trigged by drusen, invades through Bruch’s membrane into the sub-RPE or sub-retinal space, a process termed choroidal neovascularization (CNV). Current therapy for nAMD inhibits angiogenesis by blocking vascular endothelial growth factor (VEGF) via intravitreal injections, which improve vision by 5–10 letters [1]. However, frequent injections are expensive, include the risk of endophthalmitis, and 15% of patients lose vision despite monthly treatment [2]. Therefore, an unmet need exists for alternative therapies.

Multiple complement genes are genetically linked to AMD pathogenesis [3–5]. Drusen are comprised of several features, including complement factors and components [6], which are chemotactic for innate immune cells like macrophages [7]. Macrophages are detectable in surgically excised CNV membranes from patients [8], and loss of macrophages reduces experimental CNV area in mice [9]. Furthermore, macrophage depletion results in choroidal vascular atrophy in mice [10]. These data implicate macrophages in steady state vascular homeostasis and pathological angiogenesis during CNV and nAMD.

Macrophages are heterogeneous cells with distinct origins. Recent studies have identified macrophage populations derived from erythromyeloid progenitors (yolk sac, fetal liver) and bone marrow-derived peripheral blood monocytes [11]. In the central nervous system, specialized macrophage populations have been described including long-lived, yolk sac-derived microglia, and monocyte-derived choroid plexus macrophages [12]. Similarly, the eye contains distinct macrophage populations, including long-lived, yolk sac-derived retinal microglia, and blood monocyte-derived choroidal macrophages [13]. However, despite these similarities, far less is known about ocular macrophage heterogeneity and its impact upon function.

In this report, we investigated ocular macrophage heterogeneity, the origin of macrophage subsets, and their contribution to nAMD pathogenesis. Using multi-parameter flow cytometry, we identified 4 macrophage subsets in mice: microglia, MHCII^−^, MHCII^+^CD11c^−^, and MHCII^+^CD11c^+^ macrophages. Using the laser-induced CNV model, C-C chemokine receptor 2 (CCR2) knockout mice, and fate mapping studies, we demonstrated that each macrophage subset has distinct origins. After laser injury, MHCII^−^ macrophages originate from classical monocytes and an expansion of tissue resident macrophages, MHCII^+^CD11c^−^ macrophages are entirely derived from classical monocytes, and MHCII^+^CD11c^+^ macrophages are derived from classical monocytes, non-classical monocytes, and an expansion of tissue resident macrophages. At steady state, microglia and MHCII^−^ macrophages are long lived, tissue resident macrophages, while MHCII^+^ macrophages are partially replenished from blood monocytes. In order to translate these findings to human, we re-analyzed recently published single-cell RNA-seq data from the human choroid [14]. Similar to our findings in mice, we identified choroidal macrophage subsets categorized based on MHCII and CD11C expression, including two MHCII^+^CD11C^−^ and three MHCII^+^CD11C^+^ populations. Gene ontology (GO) enrichment analysis found three subsets enriched for angiogenesis-related genes. These studies demonstrate the presence of macrophage heterogeneity in mice and humans, and support the concept of pro-angiogenic, pathogenic macrophages, which could be future therapeutic targets.

## Methods

### Animals

Breeding pairs of wildtype (C57BL6/J; #000664), *Ccr2*^−/−^ (B6.129S4-*Ccr2*^*tm1Ifc*^/J; 004999), *Cx3cr1*^*creER*^ (B6.129P2(C)-*Cx3cr1*^*tm2.1(cre/ERT2)Jung*^/J; 020940), and *ROSA26*^*zsGFP*^ (B6.Cg-*Gt(ROSA)26Sor*^*tm6(CAG-ZsGreen1)Hze*^/J; 007906) were obtained from Jackson Labs (Bar Harbor, ME). Wildtype and *Ccr2*^−/−^ animals used in this study were first- or second-generation crosses of parental mice. Mac^GFP^ (*Cx3cr1*^*creER/+*^; *ROSA26*^*zsGFP/+*^) mice were first-generation offspring of the parental lines above resulting in mice heterozygous for each allele. One complete litter from each breeding pair was genotyped to confirm the correct genotype and the absence of the RD8 allele (*Crb1*^−^). Genotyping services were performed by Transnetyx (Cordova, TN).

### Tamoxifen administration

Tamoxifen (T5648, Sigma-Aldrich, St. Louis, MO) was dissolved in corn oil (C8267, Sigma-Aldrich) at 20 mg/ml with shaking at 37 °C overnight. Tamoxifen solutions were stored at 4 °C for less than 1 week. Tamoxifen was administered via intraperitoneal injection (75 mg/kg body weight) twice separated by 48 h via a 25G needle. Control animals received 100 μl of corn oil using the same technique. Tamoxifen or corn oil was administered at 6–8 weeks of age.

### Laser-induced CNV

Male and female 10–12-week-old mice were treated as previously described [15]. Briefly, mice were anesthetized with ketamine/xylazine (Akorn, Lake Forest, IL). Pain control and hydration were achieved with a 1 mg/kg subcutaneous injection of Meloxicam (Henry Schein Animal Health, Melville, NY). Eyes were anesthetized and dilated, and a cover slip was coupled to the cornea with Gonak (Akorn) for slit lamp biomicroscopy and laser. Four (immunofluorescence) or eight (flow cytometry, to increase inflammatory cell numbers) focal burns (75 μm, 110 MW, 100 ms) were administered in each eye using a 532 nm argon ophthalmic laser (IRIDEX, Mountain View, CA) via a slit lamp (Zeiss, Oberkochen, Germany).

### Immunofluorescence

Eyes were treated as previously described [15]. Briefly, mice were sacrificed 3 days (IBA1 and ICAM-2) or 2 weeks (ICAM-2) after laser-induced CNV. Enucleated eyes were fixed for 1 h in 1% paraformaldehyde (#15713-S, Electron Microscopy Sciences, Hatfield, PA) at room temperature. Eyes were washed in PBS and dissected to remove conjunctiva, cornea, iris, ciliary body, lens, and retina leaving a posterior eye cup of RPE, choroid, and sclera. Eye cups were blocked in Tris-buffered saline (TBS) + 5% Donkey serum (S30, Sigma-Aldrich), then treated with an anti-IBA1 and/or anti-ICAM-2 primary antibody (both 1:500, Table 1), and Alexa Fluor 647-conjugated anti-rabbit and/or Alexa Fluor 488-conjugated anti-rat secondary antibody (Table 1). Pictures were captured on a Ti2 widefield microscope (Nikon, Melville, NY). Area was analyzed using ImageJ after masking of images.

**Table 1:** Antibodies used in this study

Antibody	Fluorophore	Clone	Usage	Manufacturer
Rat anti-mouse CD16/CD32	N/A	2.4G2	F_c_ block	BD Biosciences
Mouse anti-mouse CD64	PE	X54-5/7.1	Eye	BioLegend
Hamster anti-mouse CD11c	BV 421	HL3	Eye^a^	BD Biosciences
Rat anti-mouse Ly6G	PE-CF594	1A8	Eye	BD Biosciences
Mouse anti-mouse NK1.1	PE-CF594	PK136	Eye	BD Biosciences
Rat anti-mouse Siglec F	PE-CF594	E50-2440	Eye	BD Biosciences
Rat anti-mouse B220	PE-CF594	RA3-6B2	Eye	BD Biosciences
Rat anti-mouse CD8	PE-CF594	53-6.7	Eye and blood	BD Biosciences
Rat anti-mouse CD4	PE-CF594	RM4-5	Eye and blood^a^	BD Biosciences
Rat anti-mouse MHC II	AlexaFluor 700	M5/114.15.2	Eye	BioLegend
Rat anti-mouse CD11b	APC-Cy7	M1/70	Eye^a^	BD Biosciences
Rat anti-mouse CD45	PE-Cy7	30-F11	Eye and blood^a^	BD Biosciences
Rat anti-mouse Ly6G	PerCP-Cy5.5	1A8	Blood^a^	BD Biosciences
Rat anti-mouse CD11b	eFluor 450	M1/70	Blood^a^	Invitrogen
Rat anti-mouse CD19	APC	1D3	Blood^a^	BD Biosciences
Mouse anti-mouse NK1.1	AlexaFluor 700	PK136	Blood	BD Biosciences
Rat anti-mouse CD115	PE	AFS98	Blood	Invitrogen
Rat anti-mouse CD19	PE	1D3	Compensation	BD Biosciences
Rat anti-mouse CD19	AlexaFluor 700	1D3	Compensation	BD Biosciences
Fixable viability dye	eFluor 506	N/A	Eye^a^	Invitrogen
Rat anti-mouse CD102 (ICAM2)	N/A	3C4(mIC2/4)	Immunofluorescence	BD Biosciences
Rabbit anti-mouse IBA1	N/A	019-19741	Immunofluorescence	Wako
Donkey anti-rat (H+L)	AlexaFluor 488	N/A	Immunofluorescence	Invitrogen
Donkey anti-rabbit (H+L)	AlexaFluor 647	N/A	Immunofluorescence	Invitrogen

List of antibodies, fluorophores, manufacturers, and clones. ^a^Antibody also used for compensation setup

### Flow cytometry of whole eyes

Experiments were performed as described [16]. Briefly, mice were sacrificed and eyes enucleated into HBSS. Animals were not perfused as we previously demonstrated no difference in macrophage numbers at steady state or after laser injury with or without systemic perfusion [16]. Eyes were cleaned of optic nerve, extraocular muscles, orbital tissue, and conjunctiva. Whole mouse eyes including cornea, sclera, iris, ciliary body, vitreous, retina, and choroid, were minced into small pieces. Eye pieces were further mechanically and chemically digested before passing through a fine mesh filter to obtain a single cell suspension. Cell suspensions were stained for live cells and washed. Cell suspensions were blocked and stained with cell surface antibodies found in Table 1. Both eyes were pooled from one mouse to determine cells per mouse, using counts beads as previously described [16]. For dissected iris, choroid, and retina, only chemical digestion was performed without mincing of tissue or mechanical digestion. After passing the tissue through a fine mesh filter, the dissected and whole eye specimens were treated identically. Two unlasered mice in tamoxifen studies were pooled to increase macrophage numbers at steady state. Samples were run on a modified LSRII (BD Biosciences, San Jose, CA) and analyzed using FlowJo v10.

### Flow cytometry of peripheral blood

Blood from sacrificed male animals was obtained with a 30G heparin needle via cardiac puncture. Samples were placed in EDTA tubes (Sarstedt, Numbrecht, Germany) to prevent clotting. In 5 ml polystyrene tubes, 1 μl of F_c_ block was incubated with 90 μl of blood for 15 min at room temperature. To this mixture, 10 μl of an antibody cocktail (Table 1) was added. Samples were vortexed gently and incubated for 30 min at 4 °C. Following incubation, 1.5 ml of lysis and fixation buffer (FACSLyse, BD Biosciences) was added. Samples were vortexed gently and placed for 10 min in the dark. Following lysis, 2 ml of MACS buffer (Miltenyi Biotec, Auburn, CA) stopped the reaction. Samples were centrifuged at 350×*g* at 4 °C for 10 min. Pellets were resuspended in 500 μl MACS buffer and moved to 1.2 ml polypropylene tubes. This wash was repeated 3 times before resuspending in a final volume of 150 μl MACS buffer. Samples were run on a modified LSRII and analyzed using FlowJo v10.

### Bioinformatics

Gene expression data (.tsv files) from human choroidal samples were downloaded from the GEO database (GSE135922). Data was imported into Seurat v3 [17, 18]. The FindIntegrationAnchors followed by the IntegrateData functions (dims 1:50) were used to integrate the data into one data set and perform batch corrections. The data were rescaled (ScaleData function), and principal component analysis (PCA) was performed (RunPCA, npcs = 50). The Elbow Plot technique was used to identify 19 significant principal components (PCs). Cells were clustered using FindNeighbors (dims = 1:19) followed by FindClusters (resolution = 0.4). The RunUMAP function was used to visualize the cell clusters. Differential expression and cell identification were performed using FindAllMarkers (min.pct = 0.25, logfc.threshold = 0.25). The DotPlot function was used to visualize gene expression.

The leukocyte subset was created by making a subset of the leukocyte clusters, followed by scaling the data (ScaleData), and PCA analysis (RunPCA). The Elbow Plot technique was again used to identify 12 significant PCs. The cells were clustered using FindNeighbors (dims = 1:12) followed by FindClusters (resolution = 0.4). Clusters were visualized using RunUMAP. Differential expression and cell identification were again performed using FindAllMarkers (min.pct = 0.25, logfc.threshold = log(2)). The DotPlot function was used to visualize gene expression. Enrichment in nAMD patients was performed by DimPlot (group.by=orig.ident) and table(eye.integrated@active.ident, eye.integrated$orig.ident). Gene expression was visualized using the VlnPlot function.

Gene ontology (GO) enrichment analysis was performed on upregulated and downregulated genes independently using a fold change cut-off = > 2.0 or < 0.5, and adjusted *p* value < 0.001. GOrilla was used for GO enrichment [19, 20], using a background of genes expressed only in Mac-A, Mac-B, Mac-C, Mac-D, and Mac-E. REVIGO was used to remove dispensable GO terms [21]. All GO terms are visualized in Fig. 7 that met a dispensability cutoff of < 0.05, enrichment > 5-fold, number of genes (b) > 2, and false discovery rate (FDR) *q* value < 0.05.

### Statistical analysis

Comparisons for CNV area were made by Mann-Whitney test due to non-parametric data distribution. Flow cytometry comparisons of macrophage numbers were made using the Brown-Forsythe and Welch ANOVA followed by Dunnett’s T3 multiple comparison test due to unequal variances between unlasered and lasered mice.

## Results

**Fig. 1:**
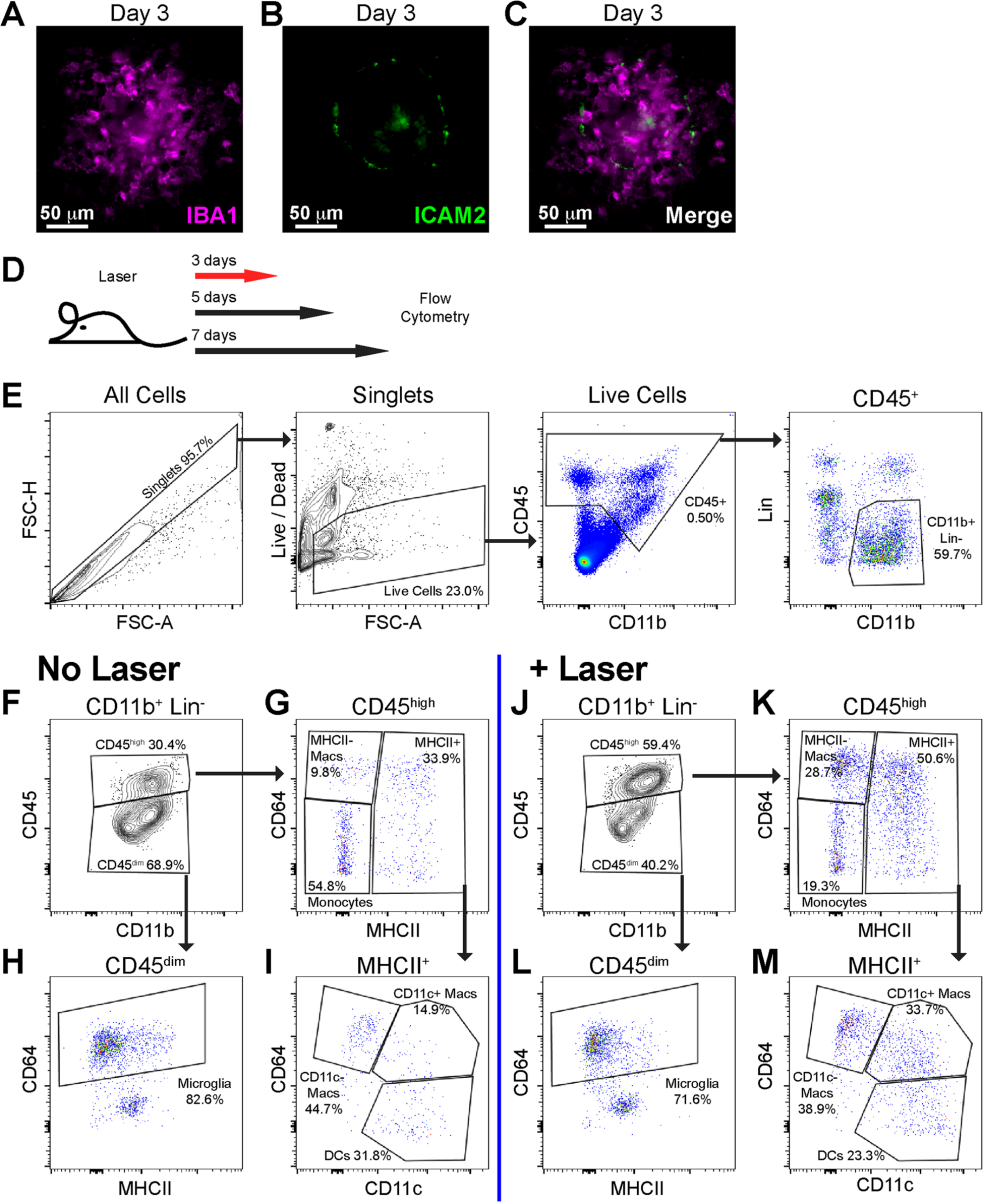
Laser CNV model and flow cytometry gating strategy for mononuclear phagocytes. IBA1 (**a**), ICAM-2 (**b**), and merged (**c**) images of a flat-mounted choroidal lesion on day 3. **d** Schematic of experimental design. Red arrow indicates that the representative plots in **e**, and **j**–**m** are from day 3 after laser injury. **e** Initial gating strategy demonstrating identification of singlet cells (left), delineation of live cells with removal of debris and count beads (middle left), gating of CD45^+^ cells (middle right), and selection of CD11b^+^, Lin^-^ cells (Lin = CD4, CD8, B220, NK1.1, SiglecF, Ly6G). **f**–**m** Gating strategy for identification of monocytes, dendritic cells (DCs), microglia, MHCII^−^, MHCII^+^CD11c^−^, and MHCII^+^CD11c^+^ macrophages from unlasered (**f**–**i**) and laser treated (**j**–**m**) mice. **f**, **j** Separation of CD45^High^ from CD45^Dim^ cells. **h**, **l** Delineation of microglia as CD45^Dim^CD64^+^MHCII^Low^ cells. **g**, **k** CD45^High^CD64^+^MHCII^−^ macrophages (MHCII^−^ Macs) are increased with laser. **i**, **m** Increased CD45^High^CD64^+^MHCII^+^CD11c^−^ (CD11c^−^ Macs) and CD45^High^CD64^+^MHCII^+^CD11c^+^ macrophages (CD11c^+^ Macs) with laser treatment

**Fig. 2:**
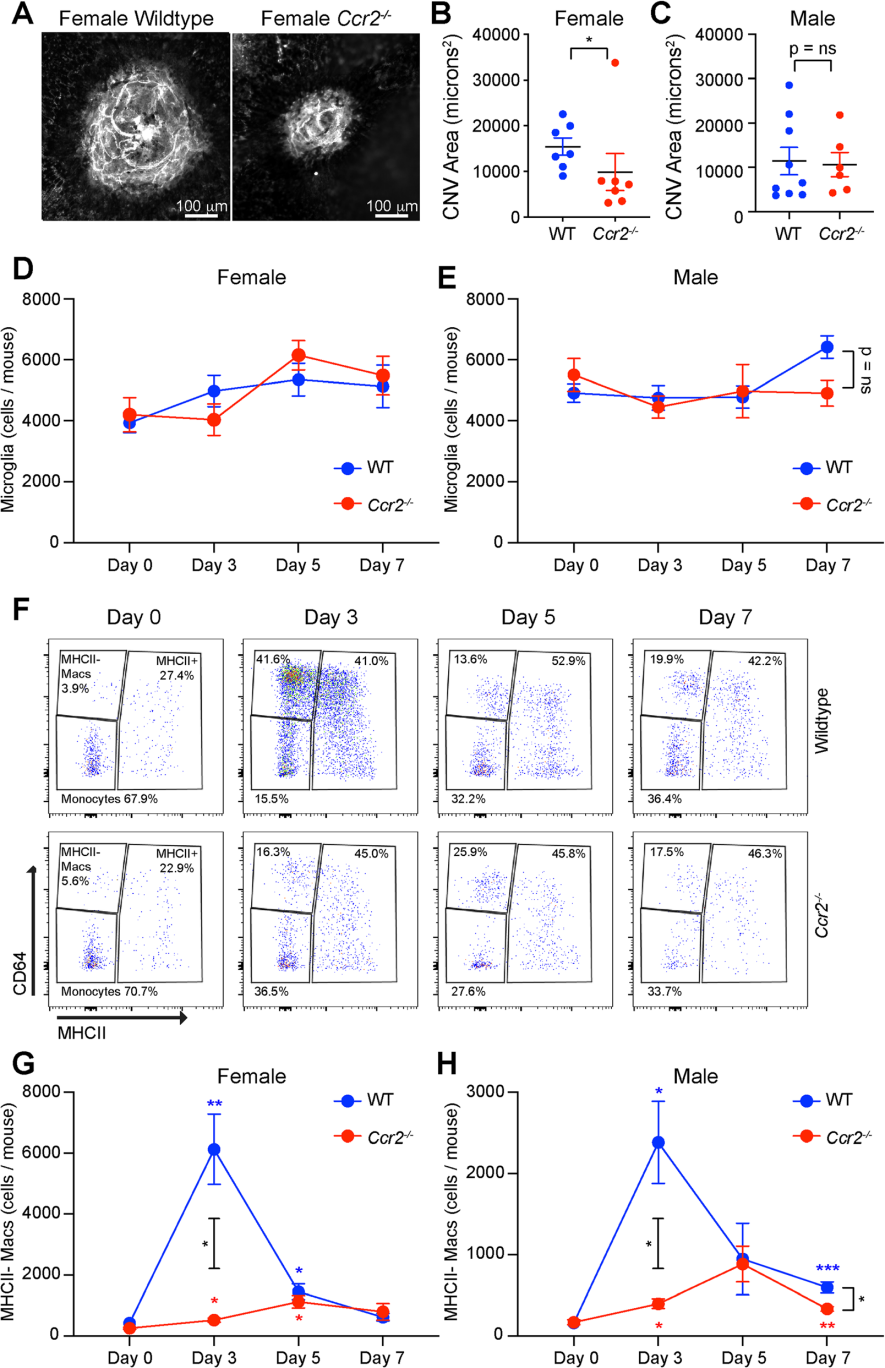
Reduced MHCII- macrophage numbers in *Ccr2*^−*/*−^ mice after laser injury. Representative ICAM2 immunofluorescence imaging (**a**) and quantitative analysis of CNV area for female (**b**) and male (**c**) wildtype (WT) vs. *Ccr2*^−*/*−^ mice. Microglia numbers were not changed by laser or genotype in female (**d**) or male (**e**) mice. **f** Representative flow cytometry pseudocolor plots from wildtype and *Ccr2*^−*/*−^ female mice from day 0, day 3, day 5, and day 7. MHCII^−^ macrophage numbers peaked in WT mice on day 3 and were blunted in *Ccr2*^−*/*−^ female (**g**) and male (**h**) mice. *p < 0.05; **p < 0.01; ***p < 0.001. Colored asterisk = significant difference from day 0 within genotype (blue = WT, red = *Ccr2*^−*/*−^ mice). Black asterisk = significant difference between genotypes on day 3 or day 7. *N* = 8–11 per group in both male and female mice. CNV was compared using the Mann-Whitney test (**b**, **c**). Macrophage numbers were compared using the Brown-Forsythe and Welch ANOVA followed by Dunnett’s T3 multiple comparison (**d**, **e**, **g**, **h**)

We used the experimental murine laser-induced CNV model to investigate macrophage heterogeneity in angiogenesis. This model triggers robust mononuclear phagocyte infiltration (IBA1^+^ cells, Fig 1a, c) with minimal neovascularization (ICAM2, Fig. 1b, c) on day 3. The angiogenic phase with many ICAM2^+^ neovessels is standardly characterized on days 7–14 (Fig. 2a). We treated 10–12-week-old male and female mice with laser and performed multi-parameter flow cytometry on day 3, 5, and 7 post laser (Fig. 1d). We dissected whole eyes and removed conjunctiva, extraocular muscles, orbital tissue, and optic nerve. Our digestion included cornea, sclera, iris, ciliary body, vitreous, retina, and choroid in order to achieve maximal rigor and reproducibility while minimizing variance created by unequal dissections. On day 3 after laser injury, we gated singlets, excluded dead cells and count beads, and selected CD45^+^, CD11b^+^, Lineage^−^ (Lin: CD4, CD8, B220, NK1.1, SiglecF, Ly6G) cells (Fig. 1e). We used CD45 expression levels to differentiate microglia (CD45^dim^) [22] from infiltrating immune cells (CD45^high^, Fig. 1f, j). We identified microglia as CD64^+^MHCII^low^ in the CD45^dim^ population (Fig. 1h, l). We delineated three infiltrating macrophage populations in the CD45^high^ population: MHCII^−^, MHCII^+^CD11c^−^, and MHCII^+^CD11c^+^ macrophage subsets (Fig. 1g, i, k, m). Fluorescence minus one controls for this flow cytometry panel were previously published [16].

The *Ccr2*^−/−^ mouse demonstrates deficient recruitment and mobilization of classical monocytes, and demonstrates reduced laser-induced CNV area [23], suggesting that classical monocyte-derived macrophages stimulate angiogenesis in the eye after laser injury. We used the *Ccr2*^−/−^ mouse model to investigate the influence of macrophage origin on each macrophage subtype. We first independently corroborated that female *Ccr2*^−/−^ mice demonstrate reduced CNV area at day 14 (Fig. 2a, b). Alternatively, male *Ccr2*^−/−^ mice showed no difference in CNV area compared to wildtype (WT) male mice on day 14 (Fig. 2c). Based upon these sex differences and the increased prevalence of nAMD in female patients [24, 25], we thoroughly investigated the number of macrophages after laser injury in male and female WT and *Ccr2*^−/−^ mice. We found that the CD45^dim^CD64^+^ putative microglia, which are yolk sac-derived, long-lived, and self-replenishing retinal macrophages, were unchanged by laser in both genotypes and sexes, as expected (Fig. 2d, e). In contrast, MHCII^−^ macrophage numbers peaked with a 14.6-fold increase in female WT mice on day 3 (*p* < 0.01, Fig. 2f, g). In *Ccr2*^−/−^ female mice, MHCII^−^ macrophage numbers increased from 257 ± 31 cells per mouse to 525 ± 67 cells per mouse (2.0-fold, *p* < 0.05 vs. day 0), which was significantly reduced compared to WT female mice on day 3 (*p* < 0.05, Fig. 2f, g). MHCII^−^ macrophages were equally increased in female WT and *Ccr2*^−/−^ mice on day 5 (*p* < 0.05 for both genotypes vs. day 0), and back to baseline on day 7 (Fig. 2f, g). In male WT mice, MHCII^−^ macrophage numbers rose 14.8-fold on day 3 (*p* < 0.05 vs. day 0), while *Ccr2*^−/−^ male mice demonstrated a 2.3-fold increase on day 3 (*p* < 0.05 vs. day 0), which was significantly reduced compared to WT mice (*p* < 0.05 between groups, Fig. 2h). On day 7, male WT mice displayed a 3.7-fold elevation (*p* < 0.001 vs. day 0), male *Ccr2*^−/−^ mice showed a 2.0-fold increase (*p* < 0.01 vs. day 0), and this was significantly different between groups (*p* < 0.05, Fig. 2h).

**Fig. 3:**
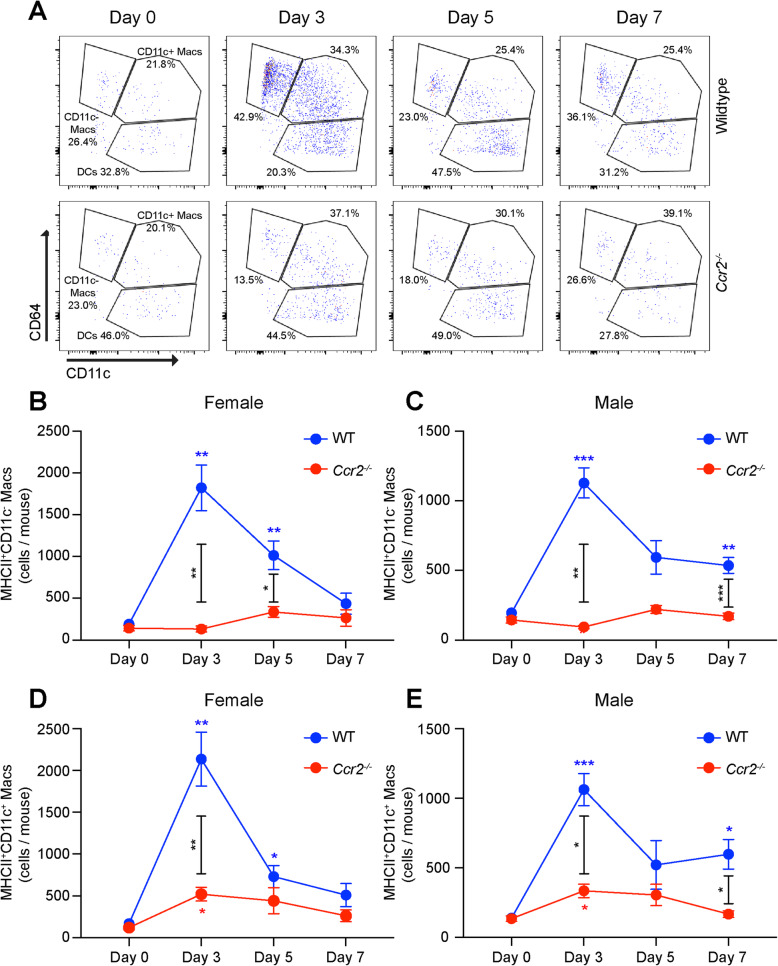
Ablated MHCII^+^CD11c^−^ and decreased MHCII^+^CD11c^+^ macrophage numbers in *Ccr2*^−*/*−^ mice in response to laser injury. **a** Representative flow cytometry pseudocolor plots from wildtype and *Ccr2*^−*/*−^ female mice from day 0, day 3, day 5, and day 7. MHCII^+^CD11c^−^ macrophage numbers peaked in WT mice on day 3 and were completely unchanged in *Ccr2*^−*/*−^ female (**b**) and male (**c**) mice. MHCII^+^CD11c^+^ macrophage numbers peaked in WT mice on day 3 and were blunted in *Ccr2*^−*/*−^ female (**d**) and male (**e**) mice. *p < 0.05; **p < 0.01; ***p < 0.001. Colored asterisk = significant difference from day 0 within genotype (blue = WT, red = *Ccr2*^−*/*−^ mice). Black asterisk = significant difference between genotypes on day 3, day 5, or day 7. *N* = 8–11 per group in both male and female mice. Macrophage numbers were compared using the Brown-Forsythe and Welch ANOVA followed by Dunnett’s T3 multiple comparison

Similar to MHCII^−^ macrophages, MHCII^+^CD11c^+^ macrophages peaked with a 12.7-fold increase on day 3 (*p* < 0.01, Fig. 3a, d) and a 4.3-fold elevation on day 5 (*p* < 0.05) in female WT mice. In *Ccr2*^−/−^ female mice, MHCII^+^CD11c^+^ macrophage numbers increased from 120 ± 27 to 521 ± 83 cells per mouse (4.3-fold, *p* < 0.05 vs. day 0), which was significantly decreased compared to female WT mice on day 3 (*p* < 0.01, Fig. 3a, d). Male WT mice demonstrated a 7.6-fold increase on day 3 (*p* < 0.001, Fig. 3e) and a 4.3-fold upregulation of MHCII^+^CD11c^+^ macrophages on day 7 (*p* < 0.05, Fig. 3e). In *Ccr2*^−/−^ male mice, MHCII^+^CD11c^+^ macrophages enlarged by 2.5-fold on day 3 only (*p* < 0.05, Fig. 3e), displaying significant reductions compared to WT on day 3 and day 7 (*p* < 0.05 between genotypes, Fig. 3e).

MHCII^+^CD11c^−^ macrophages increased 9.6-fold (*p* < 0.01 vs. day 0) on day 3 and 5.3-fold (*p* < 0.01 vs. day 0) on day 5 in female WT mice, and were completely unchanged in *Ccr2*^−/−^ female mice (Fig. 3a, b). In male mice, MHCII^+^CD11c^−^ macrophages numbers elevated 5.8-fold (*p* < 0.001 vs. day 0) on day 3 and 2.7-fold (*p* < 0.01 vs. day 0) on day 7, and were completely unchanged in *Ccr2*^−/−^ male mice (Fig. 3c). These results suggest that the increase in MHCII^+^CD11c^−^ macrophages with laser is entirely derived from classical monocytes. Alternatively, the laser injury-induced elevation of MHCII^−^ and MHCII^+^CD11c^+^ macrophages is primarily driven by classical monocytes, but there are additional contributions from non-classical monocytes and/or tissue resident macrophages in male and female mice.

**Fig. 4:**
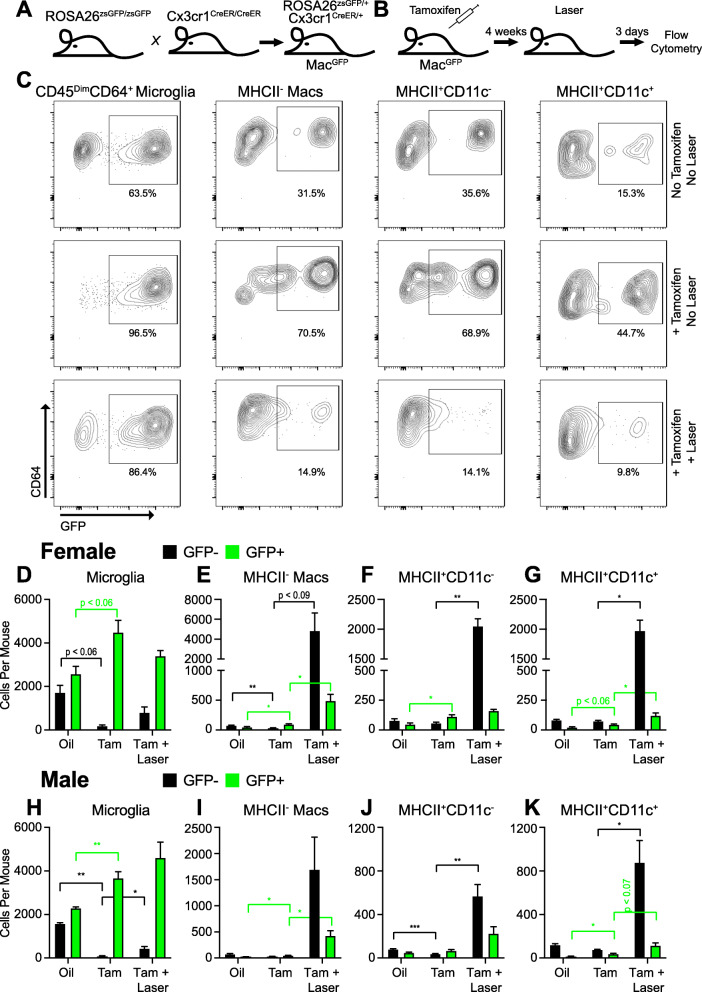
Fate mapping of macrophages subsets after laser injury. **a** Breeding strategy for generation of Mac^GFP^ mice. **b** Experimental design for fate mapping of tissue resident macrophages. **c** Representative contour plots of GFP^+^ vs. GFP^−^ macrophage subsets. **d**–**k** Quantitative analysis of GFP^+^ and GFP^−^ macrophage numbers for each subset in female (**d**–**g**) and male (**h**–**k**) mice. Oil = corn oil vehicle control, Tam = Tamoxifen. *p < 0.05; **p < 0.01; ***p < 0.001. Color = GFP− (black) or GFP+ (green) cells. *N* = 3–9 in male mice and *N* = 4–9 in female mice per group. Macrophage numbers were compared using the Brown-Forsythe and Welch ANOVA followed by Dunnett’s T3 multiple comparison

In order to investigate tissue resident macrophages, we generated *Cx3cr1*^*CreER/+*^; *Rosa26*^*zsGFP/+*^ mice (Mac^GFP^, Fig. 4a). Upon tamoxifen administration, *Cre* expression in *Cx3cr1*^+^ cells will excise the stop codon prior to the *zsGreen* gene, causing irreversible bright zsGFP expression for fate mapping [26]. Mac^GFP^ mice underwent intraperitoneal tamoxifen administration at 6 weeks of age. This strategy labeled *Cx3cr1*^+^ tissue resident macrophages and peripheral blood monocytes as GFP^+^. Peripheral blood monocytes are replenished from bone marrow after ~ 2 weeks; thus, ~ 4 weeks after tamoxifen administration, we expect peripheral blood monocytes to be GFP^−^. We performed multi-parameter flow cytometry on peripheral blood. One week after tamoxifen administration, peripheral blood monocytes (72.3%, Fig. S1E), NK cells (18.2%, Fig. S1F), and neutrophils (0.37%, Fig. S1I) demonstrated increased percentages of GFP^+^ cells compared to corn oil vehicle controls. At 4–6 weeks post-tamoxifen treatment, monocytes and all other cell types had equivalent GFP^+^ percentages compared to control mice (Fig. S1E-J). These data confirmed that the Mac^GFP^ mouse is an effective fate mapping model for tissue resident macrophages.

Using our Mac^GFP^ fate mapping model, we subjected male and female mice to tamoxifen administration at 6 weeks of age followed by laser treatment at 10–12 weeks of age (Fig. 4b). We performed multi-parameter flow cytometry on day 3 after laser injury, the peak of macrophage recruitment, in order to determine the contribution of tissue resident (GFP^+^) vs. monocyte-derived (GFP^−^) macrophages to each macrophage subset. The number of GFP^+^ microglia increased with tamoxifen compared to corn oil control in female (Fig. 4d, *p* < 0.06) and male (Fig. 4h, *p* < 0.01) mice. Similarly, the number of GFP^−^ microglia decreased with tamoxifen treatment in female (Fig. 4d, *p* < 0.06) and male (Fig. 4h, *p* < 0.01) mice. After laser injury, there was no change in GFP^+^ microglia in male or female mice (Fig. 4d, h), which was expected because laser injury of the choroid should not significantly affect retinal microglia numbers. Alternatively, GFP^+^MHCII^−^ macrophages increased from 49 ± 11 cells per mouse in the control to 96 ± 10 cells per mouse with tamoxifen (Fig. 4e, *p* < 0.05) and 490 ± 109 cells per mouse with tamoxifen + laser (Fig. 4e, *p* < 0.05 vs. tamoxifen) in female mice. This effect size (~ 400 cells) is equivalent to the increased number of macrophages observed in lasered *Ccr2*^−/−^ female mice on day 3 (Fig. 2g). GFP^−^MHCII^−^ macrophages were dramatically upregulated from < 100 ± 6–11 cells per mouse in control and tamoxifen-treated mice to 4847 ± 1805 cells per mouse in tamoxifen + laser-treated mice (Fig. 4e, *p* < 0.09). Nearly identical findings were observed in male mice. GFP^+^MHCII^−^ macrophages increased from 26 ± 1 in control male eyes to 48 ± 5 cells per mouse with tamoxifen (Fig. 4i, *p* < 0.05) and 428 ± 93 cells per mouse in laser + tamoxifen-treated mice (Fig. 4i, *p* < 0.05). The increased GFP^+^MHCII^−^ macrophage numbers were very similar to lasered *Ccr2*^−/−^ male mice, which increased from 169 ± 30 cells per mouse on day 0 to 397 ± 59 cells per mouse on day 3 (Fig. 2h, *p* < 0.05), suggesting that increased MHCII^−^ macrophage numbers with laser are mainly due to classical monocyte recruitment with a minor contribution from expanded tissue resident macrophages.

We next investigated the two MHCII^+^ macrophages subsets. The number of GFP^+^MHCII^+^CD11c^−^ macrophages increased with tamoxifen treatment (Fig. 4f, *p* < 0.05) but were unchanged with laser injury. Alternatively, GFP^−^MHCII^+^CD11c^−^ macrophages increased from 50–80 ± 8–15 cells per mouse in unlasered mice to 1599 ± 252 cells per mouse after laser injury (Fig. 4f, *p* < 0.01). Similar results were observed in male mice (Fig. 4j), confirming that all MHCII^+^CD11c^−^ macrophages are classical monocyte-derived macrophages after laser injury.

The number of GFP^+^MHCII^+^CD11c^+^ macrophages increased from 22 ± 6 cells per mouse in control to 46 ± 5 cells per mouse with tamoxifen treatment (Fig. 4g, *p* < 0.06) and 121 ± 22 cells per mouse with laser + tamoxifen treatment (Fig. 4g, *p* < 0.05) in female mice. The increased number of GFP^+^MHCII^+^CD11c^+^ macrophages was less than *Ccr2*^−/−^ female mice (120 ± 27 to 521 ± 83 cells per mouse, Fig. 3d). In male mice, GFP^+^MHCII^+^CD11c^+^ macrophage numbers expanded from 14 ± 4 in control to 38 ± 5 with tamoxifen (Fig. 4k, *p* < 0.05) and 115 ± 24 cells per mouse with tamoxifen and laser (Fig. 4k, *p* < 0.07). Again, this effect size was reduced compared to *Ccr2*^−/−^ male mice (136 ± 20 to 335 ± 48, Fig. 3e). These data suggest that increased MHCII^+^CD11c^+^ macrophages with laser injury originate from classical monocyte infiltration, tissue resident macrophage expansion, and a third source, which we suspect is the non-classical monocyte population.

**Fig. 5:**
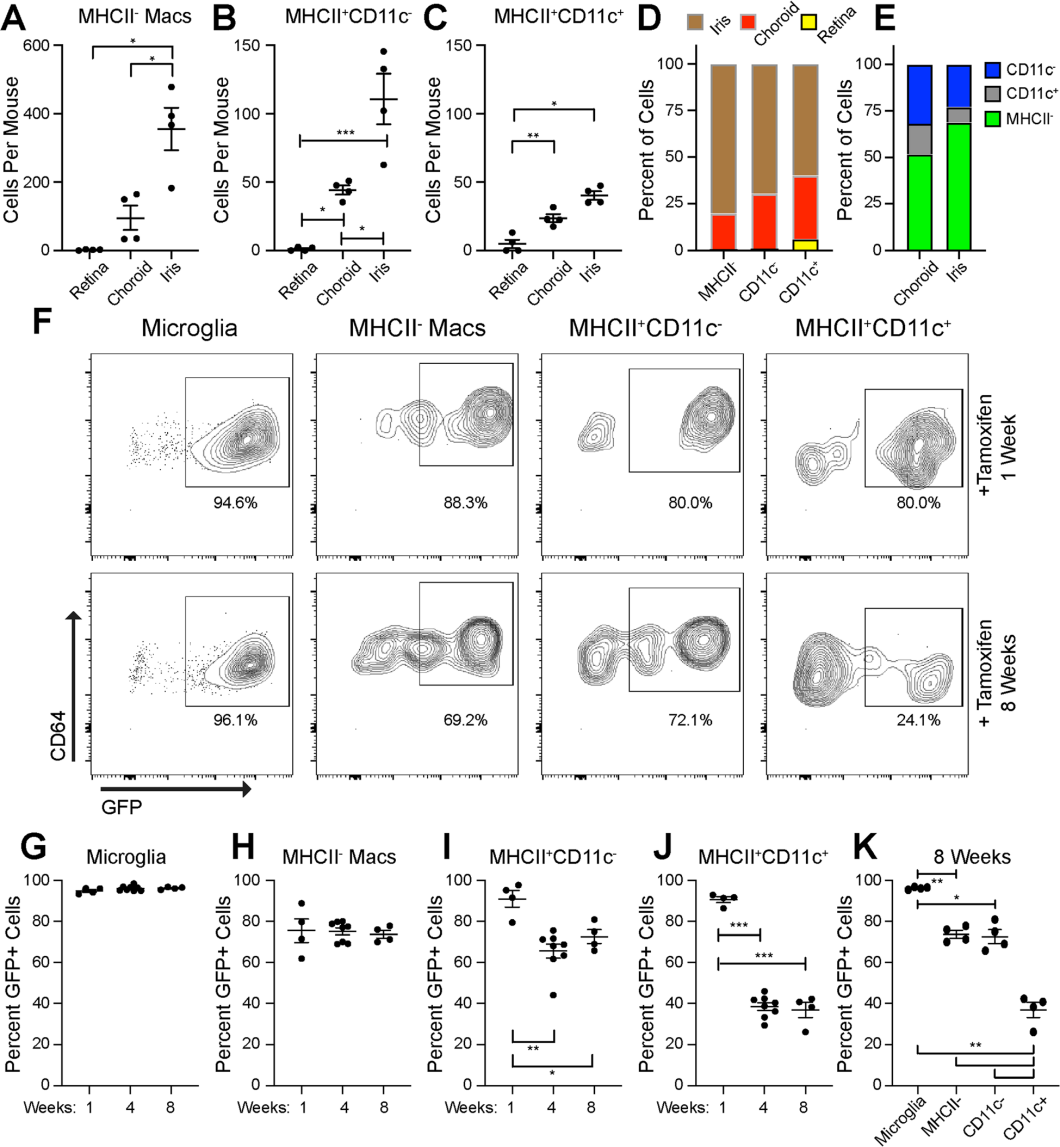
Fate mapping of steady state tissue resident macrophages. **a**–**c** Number of macrophages in each tissue at steady state. **d**, **e** Percentages of steady state macrophages by subtype (**d**) or by tissue (**e**). **f** Representative contour plots of GFP^+^ vs. GFP^−^ macrophage subsets. **g** Percent GFP^+^ microglia were unchanged from week 1 to week 8. **h** Percent GFP^+^MHCII^−^ macrophages were equivalent from week 1 to week 8. **i** Percent GFP^+^MHCII^+^CD11c^−^ macrophages were decreased at week 4 and week 8. **j** Percent GFP^+^MHCII^+^CD11c^+^ macrophages were decreased at week 4 and week 8. **k** Percent GFP^+^ macrophage subsets at week 8 were significantly different. *p < 0.05; **p < 0.01; ***p < 0.001. *N* = 4–8 per group. Macrophage numbers were compared using the Brown-Forsythe and Welch ANOVA followed by Dunnett’s T3 multiple comparison

To better understand ocular macrophages at steady state, we dissected eyes into iris, retina, and choroid fractions to determine the contribution from each tissue to each macrophage subset using multi-parameter flow cytometry. MHCII^−^ macrophages were most abundant overall, and were distributed 80% in the iris and 20% in the choroid (Fig. 5a, d). MHCII^+^CD11c^−^ macrophages were similarly divided 70% iris and 30% choroid (Fig. 5b, d). MHCII^+^CD11c^+^ macrophages were allocated 60% iris, 35% choroid, and 5% retina (Fig. 5c, d). The retina was almost entirely microglia, and the choroid contained 52% MHCII^−^, 32% MHCII^+^CD11c^−^, and 16% MHCII^+^CD11c^+^ macrophages (Fig. 5e). The iris displayed 69% MHCII^−^, 23% MHCII^+^CD11c^−^, and 8% MHCII^+^CD11c^+^ macrophages (Fig. 5e).

Next, we used the Mac^GFP^ fate mapping model to study whole eye (cornea, iris, ciliary body, sclera, vitreous, retina, and choroid) macrophage subsets at steady state. Since data from male and female mice were equivalent at steady state in both models (*Ccr2*^−/−^ and Mac^GFP^), experiments were only performed in female mice. Multi-parameter flow cytometry was performed at week 1, week 4, and week 8 post-tamoxifen administration. As expected, 95% of microglia were GFP^+^ and remained GFP^+^ through week 8, confirming that microglia are long-lived, self-replenishing tissue resident macrophages (Fig. 5f, g). Similarly, 75% of MHCII^−^ macrophages were GFP^+^, and remained GFP^+^ through 8 weeks (Fig. 5h), suggesting that steady state MHCII^−^ macrophages are self-replenishing, tissue resident macrophages. Alternatively, MHCII^+^CD11c^−^ macrophages were 91% GFP^+^ at week 1 and decreased to 65–70% GFP^+^ at week 4 (*p* < 0.01) and week 8 (*p* < 0.05, Fig. 5i). Similarly, MHCII^+^CD11c^+^ macrophages were 91% GFP^+^ at week 1 and reduced to 37–38% GFP^+^ at week 4 and week 8 (*p* < 0.001, Fig. 5j). There were no significant differences in total (GFP^−^ and GFP^+^) microglia, MHCII^−^, or MHCII^+^CD11c^+^ macrophage numbers (Fig. S2A-B, S2D). There was a small decrease in total MHCII^+^CD11c^−^ macrophages at week 1 (Fig. S2C). These data demonstrate that a portion of MHCII^+^ macrophages are replenished from peripheral monocytes, and that MHCII^+^CD11c^+^ macrophages are replenished at a higher rate than MHCII^+^CD11c- macrophages (Fig. 5k).

In order to apply our murine macrophage heterogeneity findings to humans, we re-analyzed a recently published single-cell RNA-seq data set from human RPE-choroid samples [14]. We merged the data from all 7 patients (both with and without endothelial cell [EC] enrichment), performed cell clustering with Seurat v3 [17, 18], and visualized the clusters using the uniform manifold approximation and projection (UMAP) technique (Fig S3A). We identified 21 cellular clusters, including EC, pericyte (PC), fibroblast (FB), Schwann (Schw), melanocyte (Mel), retinal pigment epithelium (RPE), retina (Ret), macrophage (Mac), T, NK, and B cells (Table S1). Additionally, we found similar EC and Schw cell heterogeneity (Fig S3B), as previously described [14].

**Fig. 6:**
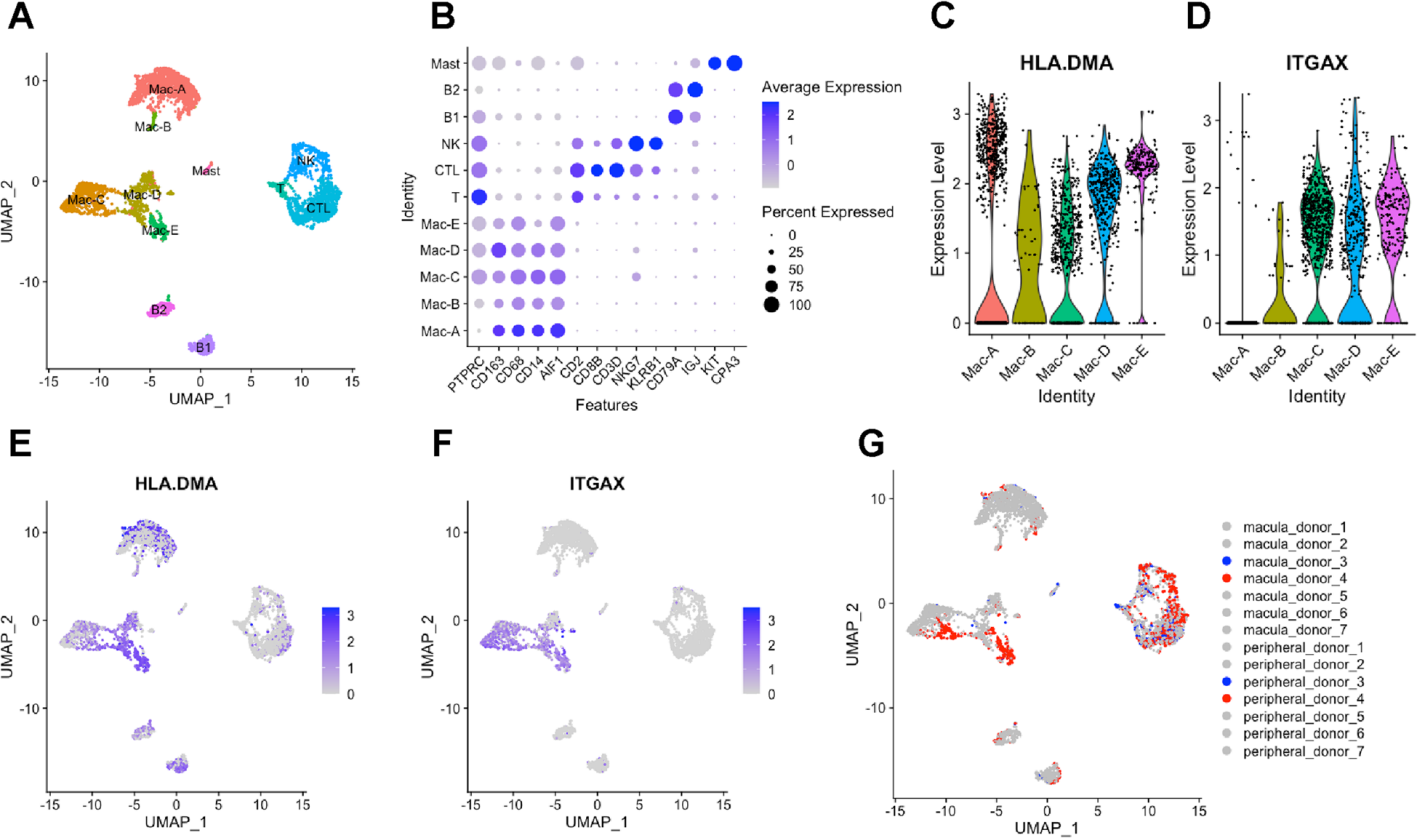
Single-cell RNA-seq analysis from human RPE-choroid samples. **a** UMAP dimension plot of 11 leukocyte clusters. **b** Dot plot of canonical macrophage, T cell, NK cell, B cell, and mast cell markers. **c**, **d** Violin plots of *HLA.DMA* (MHCII) and *ITGAX* (CD11C) gene expression in macrophage clusters. **e**, **f** Feature plots of *HLA.DMA* (MHCII) and *ITGAX* (CD11C) gene expression. **g** UMAP dimension plot grouped by donor (patients with nAMD are highlighted in blue [not EC enriched] and red [EC enriched])

In order to investigate leukocyte heterogeneity, we reclustered the leukocytes (Mac1-3, T, NK, and B1-2), and used an UMAP dimension plot to visualize the cellular gene expression. We identified 11 clusters including 5 macrophage subtypes, 3 effector NK/T cell subsets, 2 populations of B cells, and 1 group of mast cells (Fig. 6a, Table S2). Dot plot visualization identified effector NK/T cells (high *CD2*, *CD8B*, *NKG7*), B cells (*CD79A*), and previously unidentified mast cell (*KIT*, *CPA3*) clusters (Fig. 6b). The newly identified 5 macrophage clusters expressed canonical macrophage markers, including *CD68*, *CD163*, *CD14*, and *AIF1* (IBA1) markers (Fig. 6b). Mac-A and Mac-B had low *PTPRC* (CD45) expression, so we investigated microglia markers. Mac-A and Mac-B demonstrated no specific elevation of 8 microglial markers (Fig. S4), which was expected from a sample devoid of retinal tissue. To compare these subsets to our murine macrophages, we queried MHCII and CD11C expression. We found that *HLA-DMA* and other classical MHCII markers (Fig. S5) were expressed at varying levels in all 5 macrophage subsets (Fig. 6c, e). Alternatively, *ITGAX* (CD11C) was expressed in Mac-C, Mac-D, and Mac-E (Fig. 6d, f). Therefore, human choroidal macrophages share similar markers to MHCII^+^CD11c^−^ and MHCII^+^CD11c^+^ murine macrophages.

We next queried the contribution of each patient to our 5 macrophage subsets to determine if any subset is disease-associated. We found that the macula of Donor 4, who had nAMD, represented 78.3% of cells in Mac-E (Fig. 6g, Table S3). No other cluster demonstrated > 50% of cells from one patient, suggesting that Mac-E could be nAMD-associated and potentially pro-angiogenic.

**Fig. 7:**
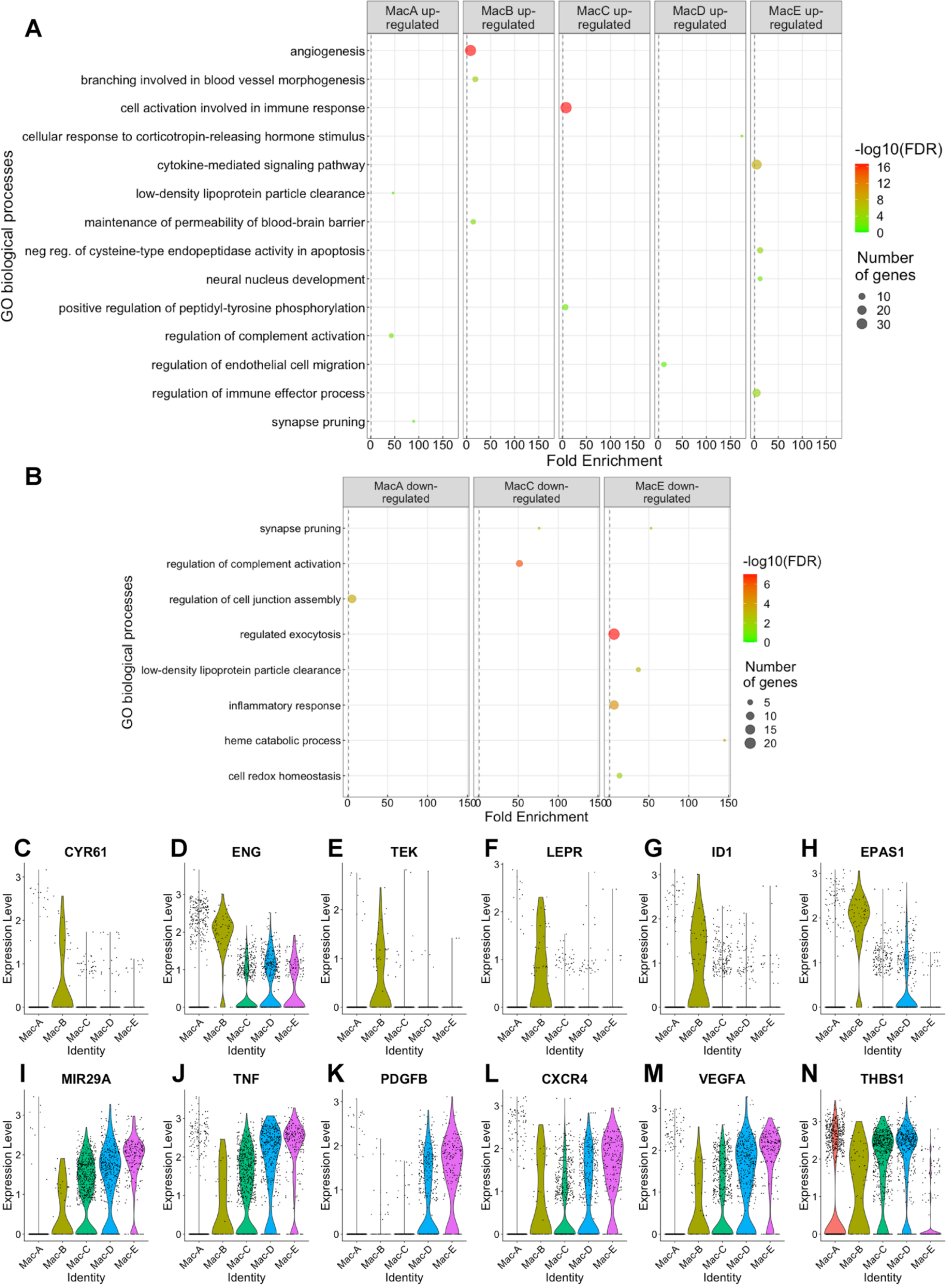
Differential expression analysis of macrophage subtypes. **a**, **b** GO term enrichment for biological process organized by upregulated (**a**) and downregulated (**b**) genes. **c**–**n** Violin plots of representative angiogenic gene expression from angiogenesis (**c**–**h**), regulation of endothelial cell migration (**i**–**k**), and cytokine-mediated signaling pathway (**l**–**n**)

Finally, we performed differential expression analysis on each macrophage subset (Table S4), followed by gene ontology (GO) enrichment to determine if any subset was enriched for vascular biology terms (Table S5). We found 8.3-fold enrichment for angiogenesis in upregulated genes from the Mac-B population (FDR *q* = 4.9 × 10^−17^, Fig. 7a). Cysteine-rich angiogenic inducer 61 (*CYR61*) is an extracellular matrix protein and endoglin (*ENG*) is a transmembrane glycoprotein. Knockdown of *Cyr61* [27] or *Eng* [28] reduce retinal neovascularization in the oxygen-induced retinopathy (OIR) mouse model. *CYR* and *ENG* were upregulated 2.1- and 3.2-fold in Mac-B, respectively (Fig. 7c, d). Tie2 (*TEK*) and *LEPR* are cell surface receptors for angiopoietin and leptin, respectively. Both macrophage-specific *Tie2* knockout mice [29] and leptin receptor antagonism [30] inhibit laser-induced CNV. *TEK* and *LEPR* expression were increased by 2.1- and 2.4-fold in Mac-B (Fig. 7e, f). *ID1* and hypoxia-inducible factor (HIF) 2alpha (*EPAS1*) are transcription factors upregulated by 2.6- and 4.9-fold, respectively, in Mac-B (Fig. 7g, h). *Id1*^−/−^ mice demonstrate reduced laser-induced CNV area and retinal neovascularization during OIR [31], and HIF-2alpha increases VEGF expression during hypoxia [32]. Therefore, Mac-B demonstrated increased expression of pro-angiogenic genes including extracellular signals, cell surface receptors, and transcription factors.

Although not as significant as the angiogenesis GO term in Mac-B, the Mac-D subset displayed 11.7-fold enrichment for regulation of endothelial cell migration (FDR *q* = 0.02, Fig. 7a). MicroRNA 29a (*MIR29A*) is expressed by macrophages and stimulates angiogenesis by downregulating anti-angiogenic genes like *TIMP3* [33]. *TIMP3* is the causative mutation for Sorsby’s macular dystrophy, which demonstrates early onset CNV, and inhibits angiogenesis by blocking VEGF signaling [34]. *MIR29A* was increased 2.0-fold in Mac-D (Fig. 7i). Tumor necrosis factor (TNF) and platelet-derived growth factor B (PDGFB) are secreted ligands that stimulate angiogenesis in the laser-induced CNV model [35, 36]. Both *TNF* and *PDGFB* were increased 2.2- and 2.1-fold in the Mac-D subset (Fig. 7j, k). Thus, Mac-D has the potential to be an additional pro-angiogenic population. Interestingly, no Mac-D upregulated genes in the regulation of endothelial cell migration GO term were shared with the Mac-B increased genes from the angiogenesis GO term.

Mac-E, the nAMD-associated population, was enriched 5.2-fold for cytokine-mediated signaling pathway (FDR *q* = 1.8 × 10^−8^, Fig. 7a). Due to our stringent dispensability cutoff, Mac-E enrichment for positive regulation of vasculature development (5.7-fold, FDR *q* = 6.6 × 10^−3^) was subsumed by cytokine-mediated signaling pathway. *MIR29A*, which was the only gene unique to the positive regulation of vasculature development GO term, was upregulated 2.7-fold in Mac-E (Fig. 7i). *TNF* and *PDGFB* were included in both GO terms and increased by 2.6- and 3.3-fold respectively in Mac-E (Fig. 7j, k). In addition, the *CXCR4* receptor, which is expressed on macrophages and is necessary for retinal and choroidal neovascularization [37], was expressed 2.5-fold more in Mac-E than other Mac subsets and included in both enriched GO terms (Fig. 7l). And finally, Mac-E demonstrated the highest *VEGFA* (2.7-fold, Fig. 7m) and lowest *THBS1* (Thrombospondin-1, 3.2-fold downregulated, Fig. 7n) expression, the two central pro- and anti-angiogenic ocular genes in CNV [38], respectively. These analyses demonstrate similar macrophage heterogeneity between mice and humans, and support the concept of pro-angiogenic choroidal macrophage subsets, which could be future therapeutic targets for nAMD.

## Discussion

**Fig. 8:**
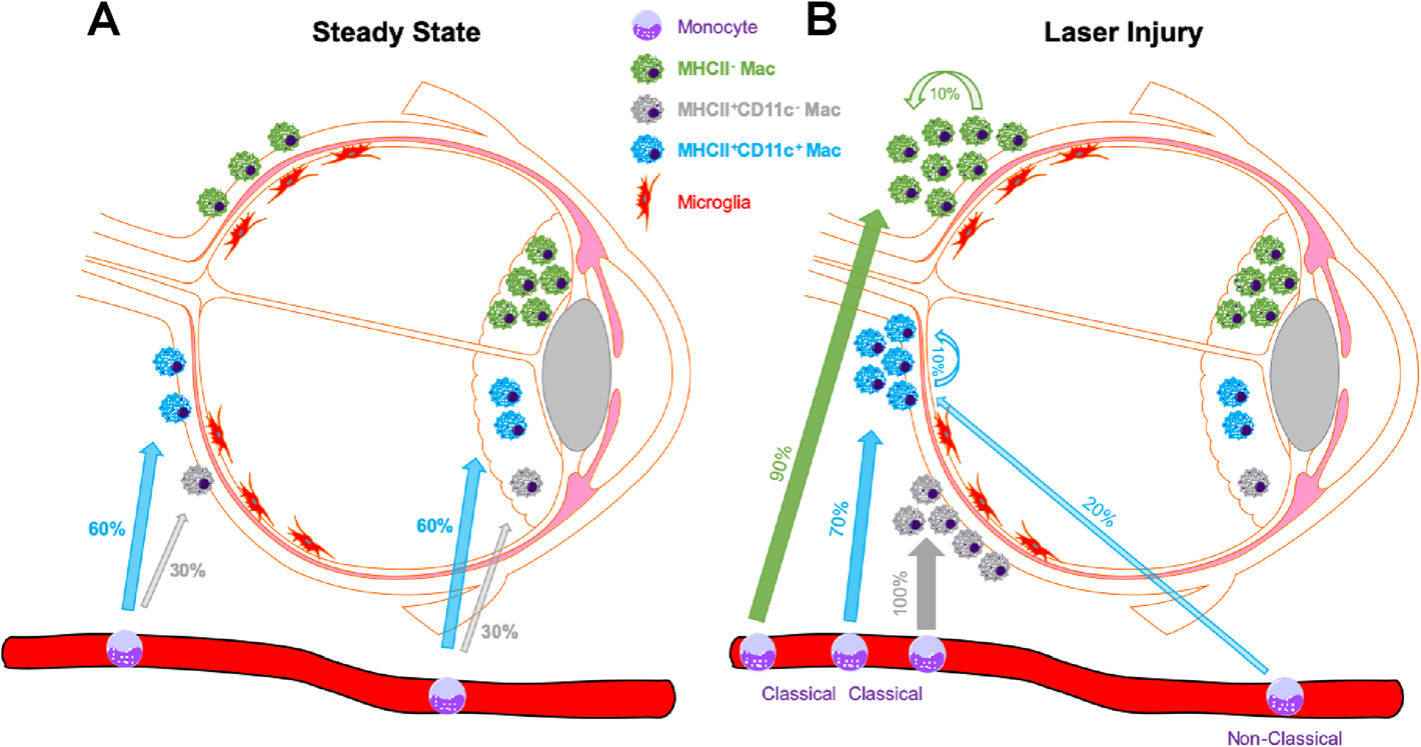
Model of ocular macrophage lineage during steady state and laser injury. **a** At steady state, microglia (red) and MHCII^-^ macrophages (green) are tissue resident, self-sustaining, and long-lived macrophages. MHCII+ macrophages (CD11c^−^ [gray] and CD11c^+^[blue]) are partially self-sustaining and partially recycled from the peripheral blood monocyte pool (numbers indicate percentage replenished from blood monocytes). **b** After laser injury, MHCII^−^ macrophages (green) are derived from classical monocytes (90%) and from expanded tissue resident macrophages (10%). MHCII^+^CD11c^−^ macrophages (gray) are 100% derived from classical monocytes. MHCII^+^CD11c^+^ macrophages are derived from classical monocytes (70%), non-classical monocytes (20%), and expanded tissue resident macrophages (10%)

In this report, we used the *Ccr2*^−/−^ mouse and the Mac^GFP^ fate mapping model to investigate how macrophage origin impacts macrophage heterogeneity at steady state and during experimental CNV. At steady state, our data suggest a model where microglia and MHCII^−^ macrophages are self-sustaining, tissue resident macrophages (Fig. 8a). In contrast, MHCII^+^ macrophages are partially replenished by blood monocytes, with MHCII^+^CD11c^+^ macrophages demonstrating a larger proportion derived from the monocyte pool (Fig. 8a). After injury, classical monocytes infiltrate the choroid and become MHCII^−^, MHCII^+^CD11c^−^, and MHCII^+^CD11c^+^ macrophages (Fig. 8b). Additionally, tissue resident macrophages expand in response to injury and contribute to the MHCII^−^ and MHCII^+^CD11c^+^ macrophage populations. Finally, a small portion of MHCII^+^CD11c^+^ macrophages are not accounted for in the *Ccr2*^−/−^ mouse or the Mac^GFP^ model, suggesting potential involvement of non-classical monocytes.

Retinal microglia are the most well-studied macrophage in the eye, and are known to be long-lived, self-replenishing tissue resident macrophages with no contribution from the monocyte pool [22]. Our data independently confirm this prior work at steady state (Fig. 5g). After laser injury, microglia are unchanged by laser or *Ccr2*-deficiency (Fig. 2d, e), suggesting that microglia numbers are relatively unaffected by laser injury to the choroid. An important limitation to our data is that because we distinguish microglia as CD45^dim^CD64^+^, activated microglia could not be included, which can express CD45^high^CD64^+^MHCII^+^ markers. Those cells could be mischaracterized as MHCII^+^CD11c^+^ macrophages, which could be an alternative explanation for the small expanded tissue resident GFP^+^MHCII^+^CD11c^+^ population (Figs. 4g and Figs. 5k). How laser injury impacts microglia gene expression and function is less understood. Microglia are known to increase VEGF expression after laser injury [39], potentially implicating microglia as pro-angiogenic. Alternatively, depletion of CD11b^+^ cells in the retina prior to laser injury, which depletes retinal microglia in addition to other cell types, has no effect upon CNV area [40], suggesting that microglia are not necessary for CNV compared to monocyte-derived macrophages. Because most prior studies do not sufficiently discriminate between microglia and infiltrating macrophages using multi-parameter flow cytometry or advanced fate mapping methods, the role of microglia during CNV remains unclear and more advanced methodologies will be needed to determine their function.

Similar to retinal microglia and synovial macrophages [41], MHCII^−^ macrophages are self-replicating with no replenishment from the peripheral monocyte pool at steady state (Fig. 5h). Since only 75% of MHCII^−^ macrophages were GFP^+^, we suspect that this population is either Cx3cr1^low^ or heterogeneously Cx3cr1^+^. Flow cytometry from ocular sub-compartments identifies that ~ 60% of iris and ciliary body macrophages and < 20% of choroidal macrophages are MHCII^−^ [42]. Our data similarly find that 70% of iris macrophages are MHCII^−^, but we find that 50% of choroidal macrophage are MHCII^−^ (Fig. 5e). The difference between our data and the prior is both mouse background (Balb/c vs. C57BL6/J) and that we distinguish dendritic cells using CD64; since dendritic cells are MHCII^+^, their inclusion will decrease the fractional abundance of MHCII^−^ macrophages in the prior study. Prior fate mapping studies show that iris and ciliary body macrophages are self-replenishing while choroidal macrophages are replenished from blood monocytes [13]. It is interesting that although 75% of MHCII^−^ macrophages reside in the iris (Fig. 5d), all MHCII^−^ macrophages are self-replenishing at steady state. This suggests that both cell surface markers and tissue microenvironment are equally important for determining cell fate.

After laser injury, MHCII^−^ macrophages increase dramatically and are significantly reduced but not abolished in *Ccr2*^−/−^ mice (Fig. 2g, h). In Mac^GFP^ mice, GFP^+^MHCII^−^ macrophage numbers grew by ~ 400 cells in male and female mice (Fig. 4e, i), which is nearly identical to the number of MHCII^−^ macrophages found in *Ccr2*^−/−^ male and female mice after laser injury. These data suggest that after laser injury, MHCII^−^ macrophages are 90% derived from classical monocytes and the remaining 10% expand from tissue resident macrophages (Fig. 8b). The fact that peripheral blood classical monocytes are ~ 80% MHCII^−^ and ~ 20% MHCII^+^ [43] supports our finding that the majority of MHCII^−^ macrophages after laser injury are derived from classical monocytes.

We identified two MHCII^+^ macrophage populations: MHCII^+^CD11c^+^ and MHCII^+^CD11c^−^. At steady state, both MHCII^+^ macrophage populations demonstrate replenishment from the peripheral monocyte pool (Fig. 5i, j). MHCII^+^CD11c^−^ macrophages are 70% tissue resident with 30% derived from peripheral blood monocytes over a 2-month period (Fig. 8a). MHCII^+^CD11c^+^ macrophages show more frequent turnover, are 30% tissue resident, and 70% replenished from monocytes. CD11c is an alpha integrin that dimerizes with CD18 to form complement receptor 4 (CR4) [44]. The function of CR4 in macrophages includes adhesion to the vasculature and is important for tissue recruitment at steady state [45], potentially explaining why MHCII^+^CD11c^+^ macrophages show more contribution from the monocyte pool. Additionally, MHCII^+^CD11c^−^ macrophages displayed higher numbers in the iris compared to the choroid (Fig. 5d, e). It is possible that the ocular sub-compartment is equally as important as CD11c expression in determining tissue resident vs monocyte-derived macrophage origin.

After laser injury, the increased MHCII^+^CD11c^−^ macrophages numbers reflect an influx of classical monocytes differentiating into MHCII^+^CD11c^−^ macrophages (Fig. 8b). This murine MHCII^+^CD11c^−^ macrophage population is similar to the human Mac-A and Mac-B subsets (Fig. 6). Interestingly, Mac-B was 8.3-fold enriched for angiogenesis genes (Fig. 7b), including ligands (*CYR61*, *ENG*), receptors (*TEK*, *LEPR*), and transcription factors (*ID1*, *EPAS1*). Although Mac-B was not as over-represented as Mac-E, 37% of cells in Mac-B were derived from the macula of a patient with nAMD. Alternatively, as macrophage depletion causes choroidal vascular atrophy [10], Mac-B could support steady state choroidal vasculature homeostasis. Considering that MHCII^+^CD11c^−^ macrophages are derived from classical monocytes, which drive angiogenesis in the choroid, and Mac-B, which is MHCII^+^CD11c^−^, is enriched for angiogenesis genes (Fig. 7b), specific anti-Mac-B therapy could be a novel therapeutic for nAMD.

After laser injury, MHCII^+^CD11c^+^ macrophages are derived from classical monocytes, expanded tissue resident macrophages, and potentially from non-classical monocytes (Fig. 8b). Human scRNA-seq analysis identifies Mac-C, Mac-D, and Mac-E as MHCII^+^CD11C^+^ macrophages (Fig. 6). These data demonstrate that MHCII^+^CD11c^+^ macrophages are both heterogeneous in murine origin after laser injury, and heterogeneous in the human choroid. Interestingly, Mac-D was enriched for regulation of endothelial cell migration, and Mac-E demonstrated enrichment for cytokine-mediated signaling pathway, which included the positive regulation of vasculature development term (Fig. 7a). In support of a CD11c^+^ disease-associated macrophage, CR4 is capable of binding Factor H [46] and is important for phagocytosis of iC3b opsonized particles [47]. Therefore, CD11c^+^ macrophages are critical for complement-mediated processes (which are strongly linked to AMD). Furthermore, Mac-E was strongly derived (78.3% of cells) from a patient with nAMD, expressed the highest *VEGFA*, and lowest *THBS1*, which are two central angiogenic factors in nAMD. The subset of classical monocyte-derived MHCII^+^CD11c^+^ macrophages are a potential key pro-angiogenic macrophage subtype.

Interestingly, the Mac-D and Mac-E upregulated genes for the regulation of endothelial cell migration and positive regulation of vasculature development GO terms were very similar to one another, but completely non-overlapping with the angiogenesis genes from Mac-B. Therefore, our analysis has uncovered two discrete macrophage-driven angiogenesis functions in the choroid.

We identified a number of interesting sex-specific differences in the laser-induced CNV model. First, female *Ccr2*^−/−^ mice demonstrated reduced CNV area, while males did not (Fig. 2a–d). This is in partial agreement with prior reports that showed ~ 75% inhibition of CNV in female *Ccr2*^−/−^ mice [23] compared to only 38% decreased CNV area in male *Ccr2*^−/−^ mice [48]. Similarly, female patients demonstrate increased prevalence of nAMD [24, 25]. When observing absolute CNV area, this difference appears to be due to larger CNV area in WT female compared to male mice. Additionally, female WT mice on average displayed twice the number of macrophages compared to males (Figs. 2 and 3). However, male WT mice showed sustained increases in MHCII^−^, MHCII^+^CD11c^−^, and MHCII^+^CD11c^+^ macrophage numbers on day 7, which was not observed in female mice. These data suggest that higher numbers of early macrophages in female mice caused larger CNV area, and/or prolonged macrophage presence decreased CNV area in male mice.

Our studies have a few important limitations. First, we used whole eye, including cornea, iris, ciliary body, vitreous, retina, and choroid for our studies. We made this decision for optimal rigor and reproducibility, and to prevent uneven ocular dissections from creating variance in our results. Because the site of injury is the choroid with some perturbations of the overlying retina, our increased macrophage numbers after laser injury most likely reflect choroidal and subretinal macrophages. However, we cannot exclude contributions from other ocular compartments. Secondly, the *Cx3cr1*^*CreER*^ transgene does demonstrate some expression without tamoxifen treatment, which is consistent with prior reports [49]. We made key comparisons between corn oil vehicle control and tamoxifen treatment, and demonstrated equivalent GFP^+^ blood monocytes 4–6 weeks after tamoxifen treatment compared to vehicle control (Fig. S1), in order to minimize noise created by the leaky *Cx3cr1*^*CreER*^ transgene. Nevertheless, rare non-specific GFP^+^ cells cannot be excluded. Lastly, we compared the number of *Ccr2*^−/−^ macrophages on day 3 vs. day 0 to the number of increased GFP^+^ macrophages on day 3 after laser treatment. We found equivalent numbers for MHCII^−^ macrophages from male and female mice, but less GFP^+^ compared to *Ccr2*^−/−^ macrophages for the MHCII^+^CD11c^+^ subset in male and female mice. From this data, we concluded that all MHCII^−^ macrophages were either derived from classical monocytes or tissue resident macrophages, and that MHCII^+^CD11c^+^ macrophages were derived from classical monocytes, tissue resident macrophages, and a third source that is either non-classical monocytes or activated microglia. However, these are two independent experiments, which may not be directly comparable, and these interpretations need to be confirmed with mice specifically deficient in non-classical monocytes.

## Conclusions

In summary, we demonstrated the presence of MHCII^−^, MHCII^+^CD11c^−^, and MHCII^+^CD11c^+^ macrophages in mouse eyes. Using the laser-induced CNV model, *Ccr2*^−/−^ mice, and Mac^GFP^ fate mapping studies, we showed that MHCII^+^CD11c^-^ macrophages were entirely derived from classical monocytes, MHCII^−^ macrophages originated from classical monocytes and an expansion of tissue resident macrophages, and MHCII^+^CD11c^+^ macrophages were derived from classical monocytes, potential non-classical monocytes, and an expansion of tissue resident macrophages. In human choroidal macrophages, we identified MHCII^+^CD11C^−^ and MHCII^+^CD11C^+^ populations. Two discrete macrophage-driven pro-angiogenic clusters were found in the MHCII^+^CD11C^−^ and MHCII^+^CD11C^+^ groups. These studies demonstrate that macrophage heterogeneity exists in the mouse and human choroid, that pro-angiogenic macrophages exist, and are a potential new therapeutic target for nAMD.

## Supplementary Information

**Additional file 1: Figure S1.** Gating strategy and analysis of peripheral blood in male Mac^GFP^ mice. (A) Gating strategy for the identification of singlet, CD45^+^ cells are shown across the top. B cells (CD19^+^, bottom right), T cells (CD4/CD8^+^, bottom right), NK Cells (CD11b^+^NK1.1^+^, bottom middle), eosinophils (CD11b^+^SSC-H^+^Ly6G^-^), and neutrophils (CD11b^+^Ly6G^+^) are delineated. Monocytes are identified from the CD11b^+^Ly6G^-^SSC-H^Low^ group (Not PMNs) as CD115^+^ and either GFP^+^ or GFP^-^ from oil (B), tamoxifen (Tam) Early (1 week, C), or Tam Late (4-6 weeks, D). Quantitative analysis of monocytes (E), NK cells (F), T cells (G), eosinophils (H), neutrophils (I), and B cells (H). * *p* < 0.05, *** *p* < 0.001. GFP^+^ cells were compared using the Brown-Forsythe and Welch ANOVA followed by Dunnett’s T3 multiple comparison.

**Additional file 2: Figure S2.** Absolute macrophage numbers at steady state in Mac^GFP^ mice. Total number (GFP^+^ and GFP^-^) of microglia (A), MHCII^−^ (B), MHCII^+^CD11c^-^ (C), and MHCII^+^CD11c^+^ (D) macrophages at week 1, week 4, and week 8 in tamoxifen-treated Mac^GFP^ mice. * *p* < 0.05, ** *p* < 0.01. Comparisons were made using the Brown-Forsythe and Welch ANOVA followed by Dunnett’s T3 multiple comparison.

**Additional file 3: Figure S3.** Singe cell RNA-seq analysis from human RPE-choroid samples. (A) UMAP dimension plot of 21 cell clusters. (B) Dot plot of canonical expression markers for each cell type. (C) Dot plot of canonical leukocyte markers confirming specific expression in macrophage (Mac), T (T cell), NK (NK cell), and B (B cell) clusters.

**Additional file 4: Figure S4.** Violin plots of microglia-specific genes. Violin plots of microglia-specific genes demonstrate no consistently increased expression of any gene in the Mac-A or Mac-B subsets.

**Additional file 5: Figure S5.** Violin plots of classical MHCII genes. Violin plots of classical MHCII genes showed that the majority of choroidal macrophages were MHCII^+^.

**Additional file 6: Table S1.** Top 20 genes for each cluster from full human choroidal sc-RNA-seq data set. Excel file of gene names, cluster, adjusted *p*-value (p_val_adj), percent of cells expressing gene (pct.1 = current cluster, pct.2 = all other clusters), fold change (avg_logFC = natural log fold change), and raw *p*-value (*p*_val).

**Additional file 7: Table S2.** Top 25 genes for each cluster of the leukocytes from human choroidal sc-RNA-seq data set. Excel file of gene names, cluster, adjusted *p*-value (p_val_adj), percent of cells expressing gene (pct.1 = current cluster, pct.2 = all other clusters), fold change (avg_logFC = natural log fold change), and raw *p*-value (*p*_val).

**Additional file 8: Table S3.** Number and percentage of cells from each patient that contributed to each leukocyte cluster. Number of cells, ratio of cells, phenotype of patient, and enrichment of sample from each human donor.

**Additional file 9: Table S4.** List of differentially expressed genes between macrophage subtypes. Excel file of gene names, cluster, adjusted *p*-value (p_val_adj), percent of cells expressing gene (pct.1 = current cluster, pct.2 = all other clusters), fold change (avg_logFC = natural log fold change), and raw *p*-value (*p*_val).

**Additional file 10: Table S4.** GO and REVIGO outputs for up- and down-regulated genes from each macrophage subtype. For GO tabs: Excel file of GO terms, description of GO term, p-value, FDR q-value, fold enrichment, number of genes expressed in macrophages (N), number of genes in GO term (B), number of differentially expressed genes (n), number of differentially expressed genes in the GO term (b), and the specific genes that were differentially expressed in the GO term. For REVIGO tabs: Excel file of GO term (term_ID), description of GO term, frequency, plotting information, log10 p-value, uniqueness, dispensability, and whether or not the term was eliminated (0 = no, 1 = yes) due to dispensability.

## Data Availability

The datasets used and analyzed for mouse studies are available from the corresponding author on reasonable request. All human data analyzed during this study are included in this published article and its supplementary information files.

## Abbreviations

nAMD: Neovascular age-related macular degeneration
AMD: Age-related macular degeneration
CNV: Choroidal neovascularization
WT: Wildtype
RPE: Retinal pigment epithelium
VEGF: Vascular endothelial growth factor
MHCII: Major histocompatibility complex II
CCR2: C-C chemokine receptor 2
GO: Gene ontology
PCA: Principal component analysis
PC: Principal component
EC: Endothelial cell
UMAP: Uniform manifold approximation and projection
PC: Pericyte
FB: Fibroblast
Schw: Schwann
Mel: Melanocyte
Ret: Retina
Mac: Macrophage
TNF: Tumor necrosis factor
PDGFB: Platelet-derived growth factor B
scRNA-seq: Single cell RNA-sequencing
CR4: Complement receptor 4
CYR61: Cysteine-rich angiogenic inducer 61
ENG: Endoglin
HIF: Hypoxia-inducible factor
MIR29A: MicroRNA 29a
THBS1: Thrombospondin-1
FDR: False discovery rate
