# Supplementary figures and images for: Ocular macrophage origin and heterogeneity during steady state and experimental choroidal neovascularization
Source: J Neuroinflammation. 2020 Nov 13;17:341. doi: 10.1186/s12974-020-02010-0 (PMC7666512; doi:10.1186/s12974-020-02010-0)

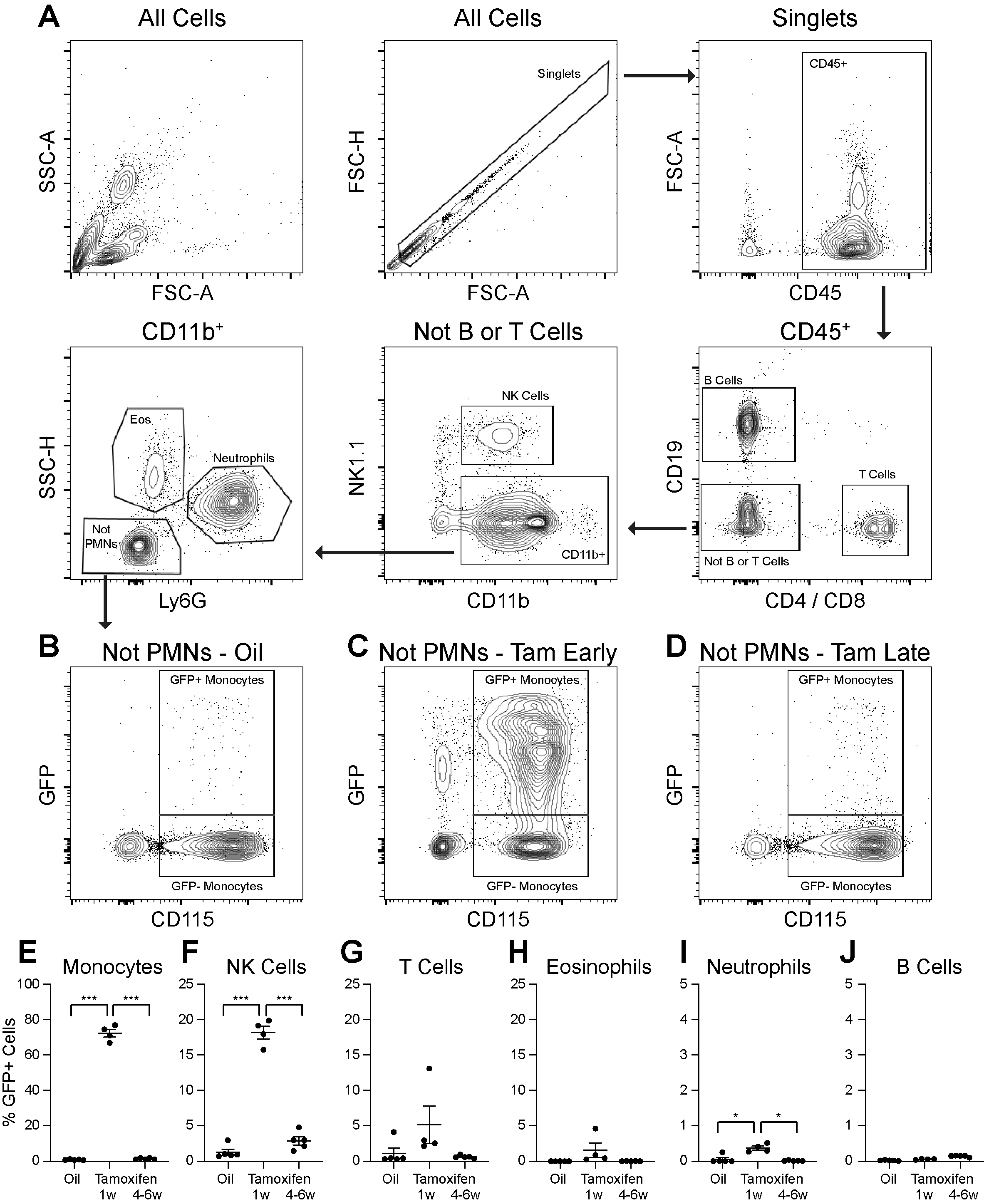

Supplement: Supplementary file 1 — Additional file 1: Figure S1. Gating strategy and analysis of peripheral blood in male MacGFP mice. (A) Gating strategy for the identification of singlet, CD45+ cells are shown across the top. B cells (CD19+, bottom right), T cells (CD4/CD8+, bottom right), NK Cells (CD11b+NK1.1+, bottom middle), eosinophils (CD11b+SSC-H+Ly6G-), and neutrophils (CD11b+Ly6G+) are delineated. Monocytes are identified from the CD11b+Ly6G-SSC-HLow group (Not PMNs) as CD115+ and either GFP+ or GFP- from oil (B), tamoxifen (Tam) Early (1 week, C), or Tam Late (4-6 weeks, D). Quantitative analysis of monocytes (E), NK cells (F), T cells (G), eosinophils (H), neutrophils (I), and B cells (H). * p < 0.05, *** p < 0.001. GFP+ cells were compared using the Brown-Forsythe and Welch ANOVA followed by Dunnett’s T3 multiple comparison. [file 12974_2020_2010_MOESM1_ESM.tif]

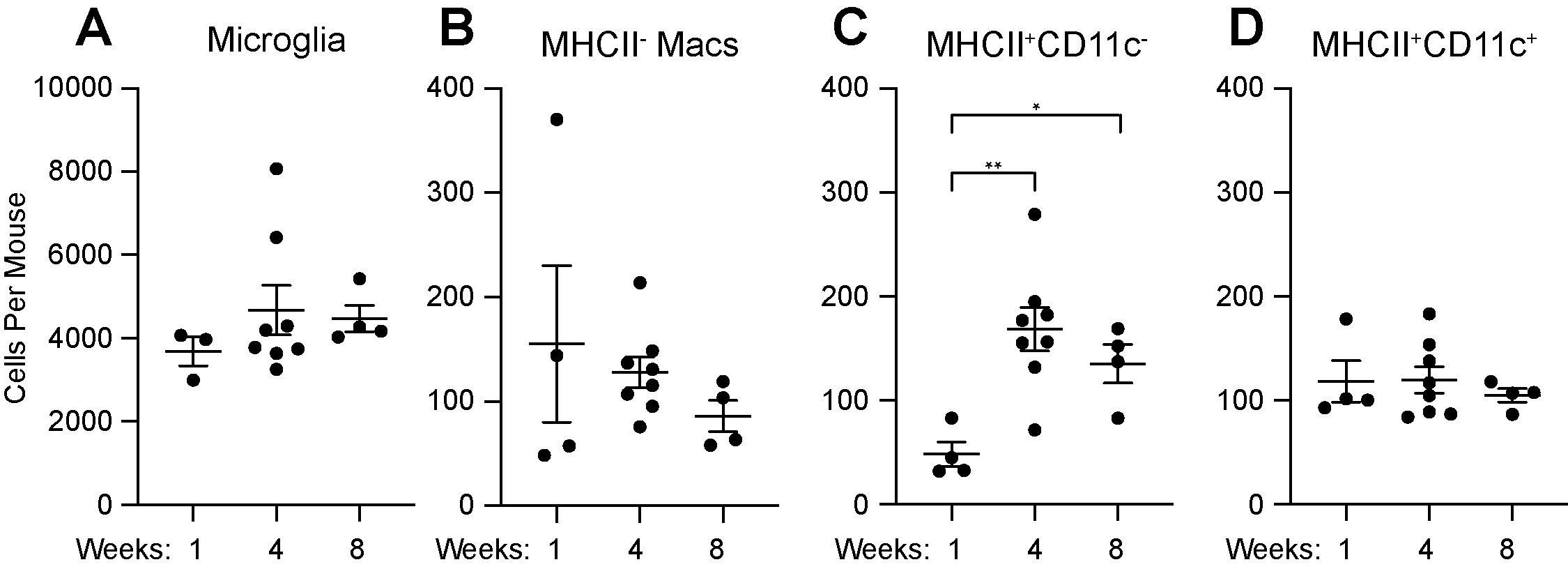

Supplement: Supplementary file 2 — Additional file 2: Figure S2. Absolute macrophage numbers at steady state in MacGFP mice. Total number (GFP+ and GFP-) of microglia (A), MHCII− (B), MHCII+CD11c- (C), and MHCII+CD11c+ (D) macrophages at week 1, week 4, and week 8 in tamoxifen-treated MacGFP mice. * p < 0.05, ** p < 0.01. Comparisons were made using the Brown-Forsythe and Welch ANOVA followed by Dunnett’s T3 multiple comparison. [file 12974_2020_2010_MOESM2_ESM.tif]

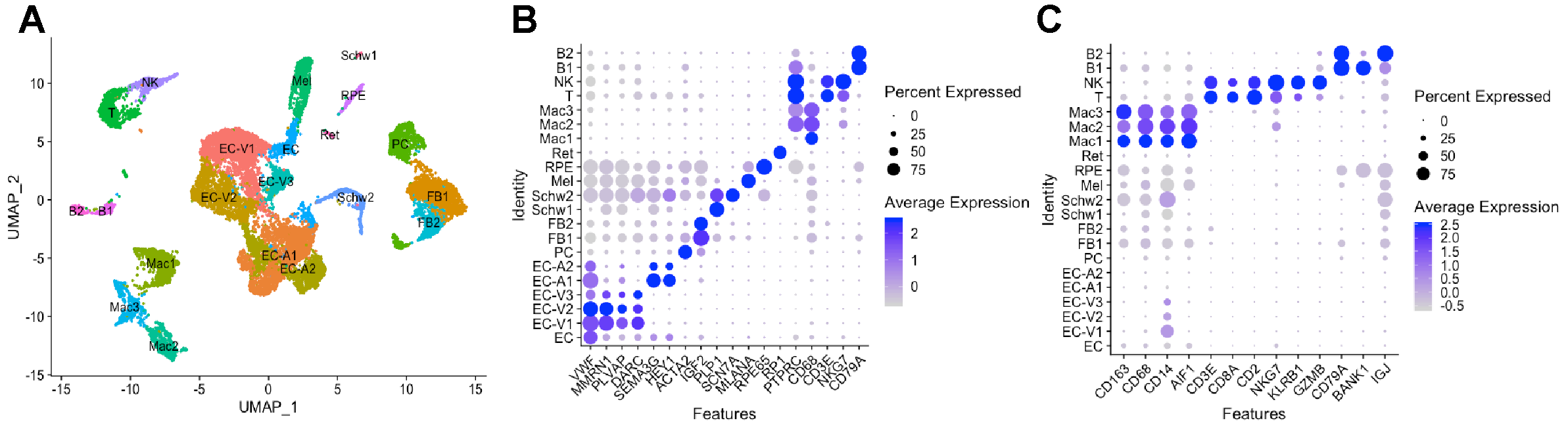

Supplement: Supplementary file 3 — Additional file 3: Figure S3. Singe cell RNA-seq analysis from human RPE-choroid samples. (A) UMAP dimension plot of 21 cell clusters. (B) Dot plot of canonical expression markers for each cell type. (C) Dot plot of canonical leukocyte markers confirming specific expression in macrophage (Mac), T (T cell), NK (NK cell), and B (B cell) clusters. [file 12974_2020_2010_MOESM3_ESM.tif]

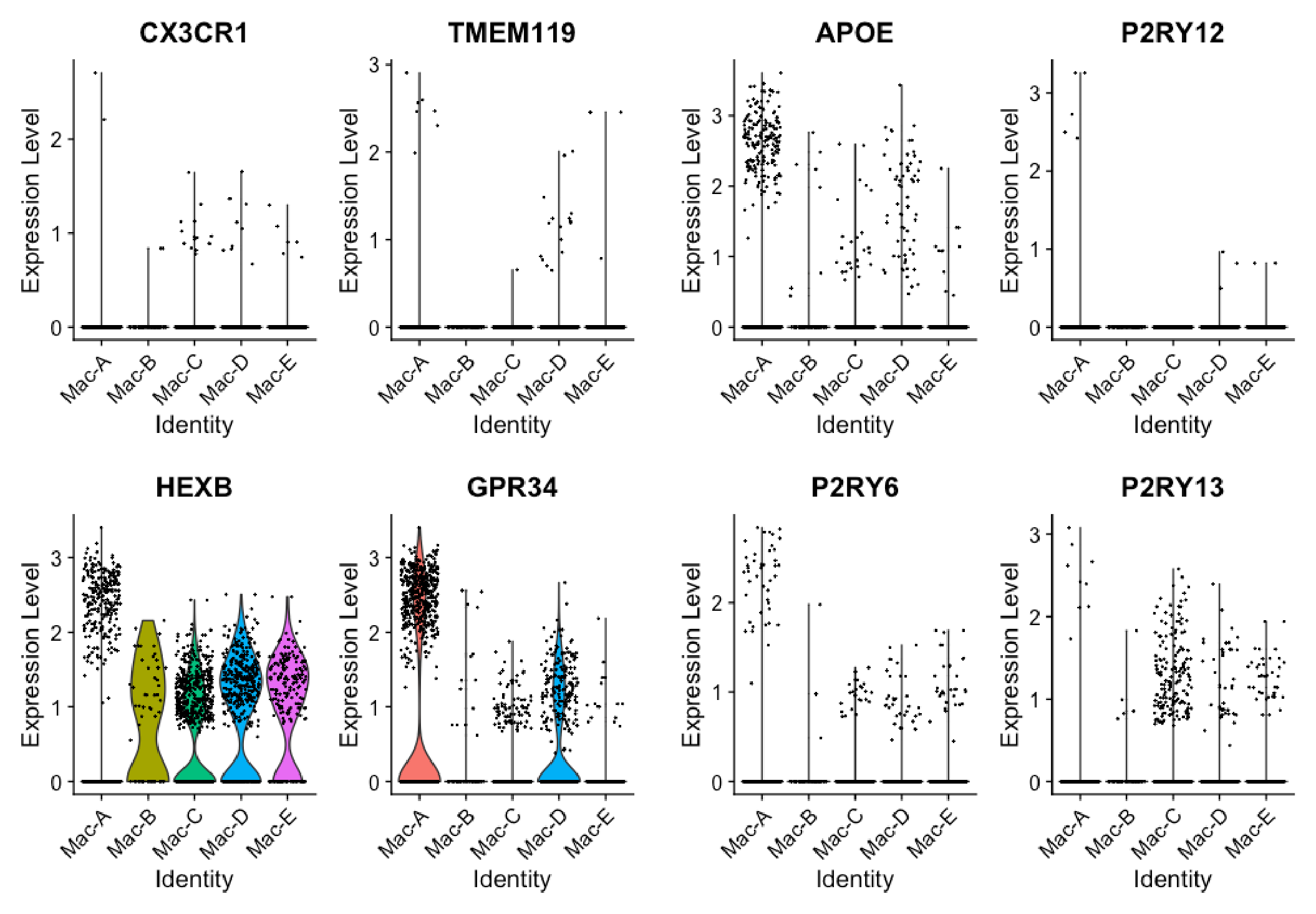

Supplement: Supplementary file 4 — Additional file 4: Figure S4. Violin plots of microglia-specific genes. Violin plots of microglia-specific genes demonstrate no consistently increased expression of any gene in the Mac-A or Mac-B subsets. [file 12974_2020_2010_MOESM4_ESM.tif]

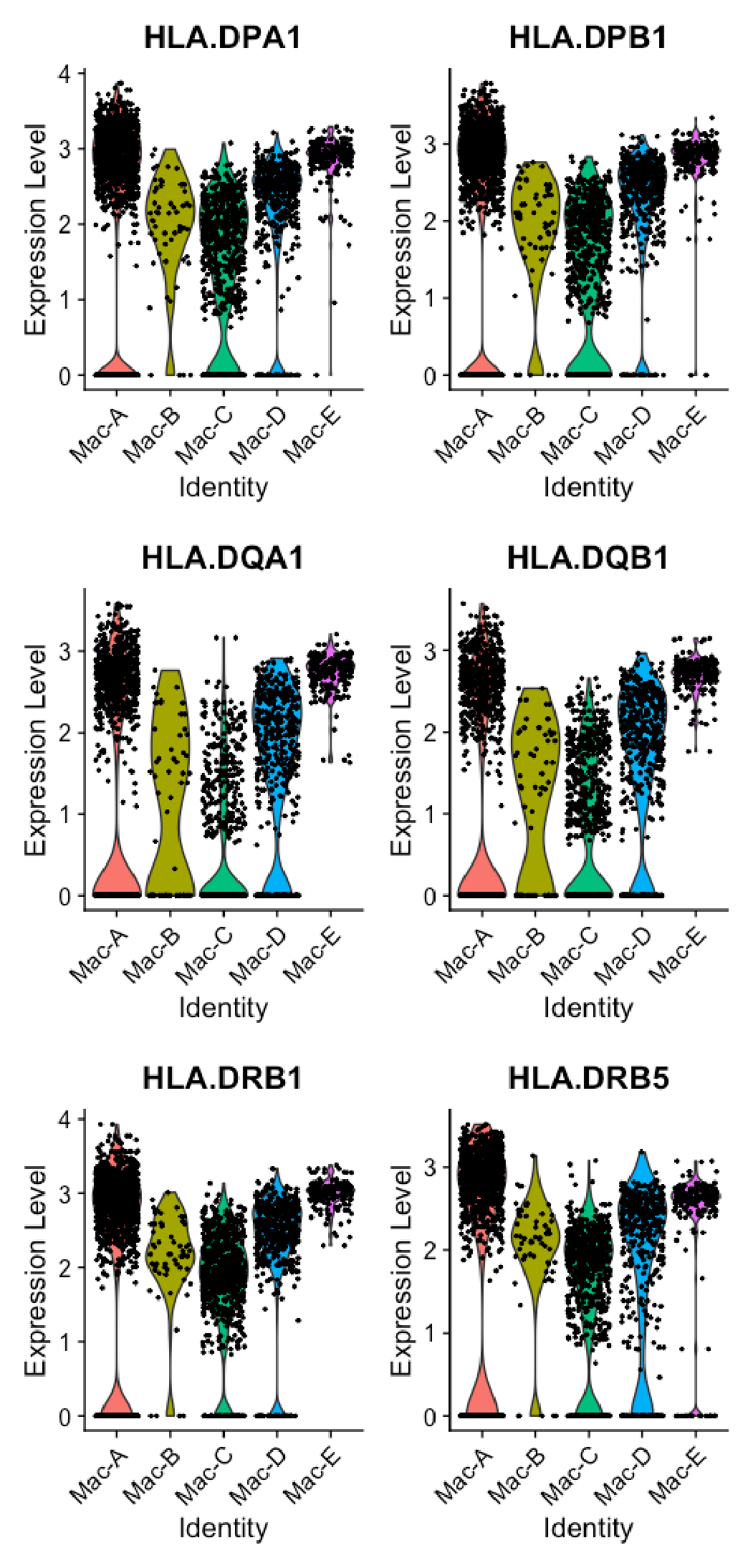

Supplement: Supplementary file 5 — Additional file 5: Figure S5. Violin plots of classical MHCII genes. Violin plots of classical MHCII genes showed that the majority of choroidal macrophages were MHCII+. [file 12974_2020_2010_MOESM5_ESM.tif]
